# Human primitive mesenchymal stem cell-derived retinal progenitor cells improved neuroprotection, neurogenesis, and vision in rd12 mouse model of retinitis pigmentosa

**DOI:** 10.1186/s13287-022-02828-w

**Published:** 2022-04-08

**Authors:** Christina Brown, Patrina Agosta, Christina McKee, Keegan Walker, Matteo Mazzella, Ali Alamri, David Svinarich, G. Rasul Chaudhry

**Affiliations:** 1 Department of Biological Sciences, Oakland University, Rochester, MI 48309 USA; 2 OU-WB Institute for Stem Cell and Regenerative Medicine, Rochester, MI 48309 USA; 3 Ascension Providence Hospital, Southfield, MI 48075 USA

**Keywords:** Retinitis pigmentosa, Retinal degenerative diseases, Retinal progenitor cells, Neuroprotection, Neurogenesis

## Abstract

**Background:**

Currently, there is no treatment for retinal degenerative diseases (RDD) such as retinitis pigmentosa (RP). Stem cell-based therapies could provide promising opportunities to repair the damaged retina and restore vision. Thus far, primarily adult mesenchymal stem cells (MSCs) have been investigated in preclinical and clinical studies, and the results have not been convincing. We applied a new approach in which primitive (p) MSC-derived retinal progenitor cells (RPCs) were examined to treat retinal degeneration in an rd12 mouse model of RP.

**Methods:**

Well-characterized pMSCs and RPCs labeled with PKH26 were intravitreally injected into rd12 mice. The vision and retinal function of transplanted animals were analyzed using electroretinography. Animals were killed 4 and 8 weeks after cell transplantation for histological, immunological, molecular, and transcriptomic analyses of the retina.

**Results:**

Transplanted RPCs significantly improved vision and retinal thickness as well as function in rd12 mice. pMSCs and RPCs homed to distinct retinal layers. pMSCs homed to the retinal pigment epithelium, and RPCs migrated to the neural layers of the retina, where they improved the thickness of the respective layers and expressed cell-specific markers. RPCs induced anti-inflammatory and neuroprotective responses as well as upregulated the expression of genes involved in neurogenesis. The transcriptomic analysis showed that RPCs promoted neurogenesis and functional recovery of the retina through inhibition of BMP and activation of JAK/STAT and MAPK signaling pathways.

**Conclusions:**

Our study demonstrated that RPCs countered inflammation, provided retinal protection, and promoted neurogenesis resulting in improved retinal structure and physiological function in rd12 mice.

**Supplementary Information:**

The online version contains supplementary material available at 10.1186/s13287-022-02828-w.

## Background

Retinal degenerative diseases (RDD), including age-related macular degeneration (AMD) and retinitis pigmentosa (RP), cause progressive and incurable vision loss [1, 2]. AMD leads to the loss of sharp and central vision due to damage to the macula that affects the central area of the retina and choroid [3], whereas RP is characterized by progressive degeneration of the photoreceptors, leading to night blindness followed by progressive loss of the visual field in daylight and ultimately blindness [4]. RP affects 1 in every 4000 people in the USA and 1 in every 5000 people worldwide [2]. In RP, mutations in the gene, *RPE65*, result in the disruption of the RPE65 protein production in the retinal pigment epithelium (RPE) [5], leading to worsened vision, night blindness, and decreased peripheral vision [6]. A mouse model, rd12, represents RP and Leber congenital amaurosis in humans, which causes blindness or severely impaired vision in adults and children, respectively [7]. The mutation responsible for vision loss in the rd12 model is a nonsense mutation within the *Rpe65* gene [8]. Using this animal model, we have previously demonstrated the therapeutic potential of embryonic stem cells (ESC)-derived neuroprogenitors [9]. Transplantation of pluripotent stem cells, ESC- and induced pluripotent stem cell (iPSC-)derived retinal progenitors, have also been reported to preserve visual function upon injection into Royal College of Surgeons (RCS) rats as well as rd1 and rd12 mice, respectively [10–12]. Because pluripotent cells can cause teratomas and pose moral and ethical concerns, growing interest has been shown to explore other options such as mesenchymal stem cells (MSCs) for cell therapy.

MSCs exert their therapeutic effect in part by secreting trophic factors that protect cells or promote their survival and transplantation of mouse bone marrow (BM)-MSCs delayed retinal degeneration in an animal model of RDD [13]. Mouse BM-MSCs transplanted into rhodopsin knockout mice integrated into the RPE and the neuroretina, which lead to prolonged photoreceptor survival [14]. Human (*h*) BM-MSCs injected into the subretinal space of a retinal dystrophy rat model showed significant and extensive photoreceptor rescue in transplanted eyes [15]. On the other hand, tail vein injection of rat MSCs preserved visual function in a rat model of RP [16]. Interestingly, transplantation of the combination of hBM-MSCs and fetal retinal progenitor cells (RPCs) into the subretinal space of a rat model of RP maintained retinal function based on the electroretinogram (ERG) results much better than when they were individually transplanted [17]. However, these findings are difficult to translate in humans, mainly because of the invasive procedures required to isolate these cells. In addition, adult MSCs have limited proliferation and differentiation potential [18]. Furthermore, adult MSCs undergo genetic changes depending on donor age and environmental stress exposure [19]. Recently, human umbilical cord (hUC) MSCs have been investigated for treating retinal degeneration. Human Wharton’s jelly (hWJ) MSCs provided neuroprotection for retinal ganglion cells (RGCs) and promoted axonal regeneration along the optic nerve in adult rats [20]. hWJ-MSCs also delayed loss of RGC in axotomy-induced rats [21], outer nuclear layer (ONL) in RCS rats [22], and improved the thickness of the retina and upregulated pro-survival genes [23]. hUC-MSCs were suggested to improve the retinal morphological structure [24], exert a neuroprotective effect, and rescue a significant percentage of axotomized RGCs [25].

The potential therapeutic effects of hBM-MSCs or adipose MSCs on retinal defects have been investigated in 30 clinical trials [26–29]. In some MSC transplantation studies, vision loss was halted, macular thickness increased, or visual acuity was improved [30–32]. Human RPC transplantation in RP patients led to moderate vision recovery and maintenance of the ONL thickness [33] or improved visual acuity [33]. It is important to note that most clinical trials are limited to phase I/II studies [26, 34] due to the lack of cells required for extensive clinical studies.

This study used human primitive (p) MSCs isolated from the hUC tissue, which is collected non-invasively and typically discarded after birth. These MSCs are considered primitive as they are highly proliferative, more naïve, and exhibit a low risk of host immune response due to the lack of expression of the major histocompatibility complex class II antigens. hUC-MSCs do not cause graft vs. host disease compared to adult MSCs [35]. We differentiated human primitive pMSCs isolated from perinatal tissue into RPCs and transplanted in the rd12 mice. The transplanted RPCs improved retinal structure and function. They also survived, dispersed, and integrated into various neural layers, while pMSCs homed to the RPE layer of the retina. These findings provide evidence that pMSC-derived RPCs are a promising source for cell therapy to treat RDD.

## Materials and methods

### Isolation and culturing of pMSCs

pMSCs were isolated and cultured following a published method [18, 36]. In brief, hUC tissue was obtained from consented healthy donors through the Ascension Providence Hospital, Southfield, MI, under an IAA (IAA #400244-10) approved protocol. The samples were rinsed with PBS several times to remove blood clots. The hUC was then dissected, minced, and plated into 75-cm^2^ culture flasks using growth medium containing DMEM nutrient mix F12 medium (DMEM/F12; Life Technologies, Carlsbad, CA, USA), supplemented with 10% fetal bovine serum (FBS; VWR, Radnor, PA, USA), and 5.6% of antibiotic solution (0.1% gentamicin, 0.2% streptomycin, and 0.12% penicillin); (Sigma, St Louis, MO, USA) and incubated at 37 °C in an atmosphere of 5% CO2 in a humidified incubator. When the explant cells reached 70% confluency, they were dissociated using TrypLE (Invitrogen, Waltham, MA, USA) and were considered as P0 cells. pMSCs were then passaged in new culture flasks for amplification, characterization, and cryopreservation for future studies.

### Differentiation of pMSCs into RPCs

pMSCs (P4) were induced to differentiate towards the retinal lineage using neurobasal media (Thermo Fisher Scientific, Waltham, MA, USA) containing 50 ng epidermal growth factor (EGF; PeproTech, Rocky Hill, NJ, USA), 1 µM retinoic acid (Sigma), 100 µM taurine (PeproTech), 2 mM glutamine (Sigma), 1 × B27 supplement (Thermo Fisher Scientific), and 2% FBS, and cultured for 2 weeks at 37 °C in an atmosphere of 5% CO_2_ in a humidified incubator. The differentiated cells were then characterized by flow cytometry, qRT‐PCR, and immunocytochemical staining analyses.

### Flow cytometry analysis

Cells were grown to 70% confluency and stained against RCVRN (APC labeled antibodies) (Becton Dickinson, Franklin Lakes, NJ, USA; Santa Cruz, Dallas, Tx, USA, respectively). Labeled cells were analyzed using FACS Canto II (Becton Dickinson) and Diva Software (Becton Dickinson). APC-labeled mouse IgG was used as a negative control.

### Animal studies

All animal experiments were approved by the Institutional Animal Care and Use Committee (IACUC), Providence Hospital, Southfield, Michigan (IACUC #101-16) and Oakland University, Rochester, Michigan (IACUC #19082), as well as the Institutional Biosafety Committee (IBC), Oakland University, Rochester, Michigan (IBC #2858). A total of 84 rd12 mutant mice from Jackson Laboratory (Bar Harbor, ME, USA) were used for this study. The rd12 mice were set up in breeding triads with one male and two females and received 7–9 pups per litter. C57BL/6J mice were used as controls. All mice were maintained at the Providence Hospital Animal Facility and Oakland University Animal Facility under a 12/12-h light and dark cycle.

### Cell transplantation in an rd12 mouse model

Cells were labeled with cell membrane labeling dye PKH26 (Sigma) following the manufacturer’s instructions. Six-week-old rd12 mice were anesthetized by intraperitoneally injecting 0.1 mL/10 g mixture of ketamine/xylazine [ketamine (50 mg/kg) and xylazine (7 mg/kg)]. A drop of topical anesthesia (Proparacaine Hydrochloride, Ophthalmic Solution, Alcaine, Acon, Canada) was applied to the eye before transplantation, and pupils were dilated with 1% atropine and 2.5% phenylephrine hydrochloride (Ophthalmic Solution). Using a 27-G sharp disposal needle, the eye was punctured by a blunt needle. The animals were intravitreally injected with 1 µL of DMEM containing PKH26 labeled pMSCs (2 × 10^5^) or RPCs (2 × 10^5^) through a 32-G needle, using a 10-µL Hamilton syringe. The experiments were performed in triplicates. The animals were monitored regularly for 4 or 8 weeks after cell transplantation.

### Electroretinography

To investigate the therapeutic effect of transplanted cells on the retina function, ERG analysis was performed 1 week before the transplantation of cells in all mice (n = 6/treatment) and every other week thereafter. Full-field ERGs were recorded with an Espion-III system (Diagnosys LLC, Lowell, MA, USA). ERGs of age-matched non-transplanted rd12 eyes were used as negative controls, and C57BL/6 J mice were used as positive controls. All testing was performed in a climate-controlled, electrically isolated dark room under dim red-light illumination. Mice were dark-adapted for 4 h and anesthetized with ketamine (50 mg/kg) and xylazine (7 mg/kg). Body temperature was maintained on a 37 °C warming pad. Proparacaine hydrochloride droplets were used to anesthetize the eyes, and pupils were dilated with 1% atropine and 2.5% phenylephrine hydrochloride. Methylcellulose gel (Ophthalmic Solution) was applied to the eyes, and the wire loop electrodes were placed on the cornea. Needle reference and ground electrodes were inserted into the cheek and tail, respectively. Dark-adapted mice were tested with a series of dim light flashes (0.001–25 cd s/m^2^). Eyes were then light-adapted to a constant background light for 10 min. Then the ERG response traces were averaged from a series of rapidly flickering bright light flashes (15 to 30 flashes per second at an intensity of 3 cd s/m^2^) over several seconds.

### Tracking of transplanted cells and histological analysis

The animals were killed by CO_2_ overdose, and the eyes were harvested 4 or 8 weeks post-transplantation. For paraffin embedding, eyes were fixed in 97% methanol and 3% acetic acid for 48 h at − 80 °C, transferred to − 20 °C for 4 h, and then 48 h at room temperature. Eyes were dehydrated thrice in 100% ethanol for 15 min and then transferred to xylene twice for 15 min. Eyes were embedded in paraffin and sectioned (5–10 μm thick) using a microtome. The paraffin-embedded retina was stained with hematoxylin and eosin (H&E) (Thermo Fisher Scientific) to evaluate the cellular structure of the retina. Twenty-five sections of the retina per treatment representing these sections were from the same location of the paraffin-embedded H&E-stained retina and were recorded for comparison.

For OCT embedding, eyes were fixed in 4% paraformaldehyde for 10 min, placed in 10% sucrose for 30 min, and then in 30% sucrose overnight at 4 °C. The eyes were then embedded in OCT medium (Thermo Fisher Scientific) and sectioned (5–10 μm thick) using a cryostat. The sections were observed under confocal microscopy for presences of PKH26 dye (Cy3) labeled cells. To mount for cell tracking, the retinas were flattened with 4–5 relaxing cuts, fixed in 4% paraformaldehyde, stained with anti-mouse RCVRN primary antibody, followed by staining of Alexa flour 488 secondary antibodies (Thermo Fisher Scientific) and analyzed by fluorescent images captured using a confocal microscope (NIKON Instruments Inc.).

### Immunocytochemical analysis

Cells and tissue slides were fixed with 4% paraformaldehyde for 10 min at room temperature, permeabilized with 0.5% Triton X-100 (Sigma), and blocked with 2% bovine serum albumin (Sigma) for 1 h. Cells and tissues were then stained with primary antibodies at 1:100 dilution at 4 °C overnight, followed by staining with secondary antibodies at 1:200 dilution at room temperature for 2 h. Cells were counterstained with DAPI at 1:100 dilution for 5 min at room temperature. Fluorescent images were captured using a confocal microscope (NIKON Instruments Inc, Melville, NY, USA). Fluorescent intensity was calculated using ImageJ software (NIH, Bethesda, MD, USA).

### qRT-PCR analysis

Isolation of the total cellular mRNA from cells and tissue was done using the GeneJET RNA purification kit (Thermo Fisher Scientific) following the manufacturer’s instructions. Total RNA was purified with DNase and incubated at 37 °C for 30 min using a thermocycler (Bio-Rad, Hercules, CA, USA). cDNA was synthesized using an iScript kit (Bio-Rad). qRT-PCR was performed by using Sso-Advanced Universal SYBR Green Supermix Kit (Bio-Rad) on CFX96 Real-Time System (Bio-Rad). The reference genes, GAPDH/*Gapdh* and β-ACTIN/*β-Actin*, were used to normalize the targeted genes. Each reaction was performed in triplicate. Primers were screened using primer BLAST [37] for homology. Human and mouse-specific primer sequences are listed in Additional file 1 and Additional file 2.

#### Analysis of retina thickness

The paraffin-embedded temporal retina was sectioned, and the same location of the H&E-stained retina was selected for each eye, to measure the thickness of the whole retina, ONL, inner nuclear layer (INL), and RGC by ImageJ (NIH, Bethesda, MD, USA). The retinal thickness analysis was performed in a blind manner.

#### RNA-sequencing analysis

RNA-seq analysis was performed as previously described [38]. In brief, the total RNA of the samples was isolated using the GeneJet RNA purification kit (Thermo Fisher Scientific). cDNA library was prepared for RNA-seq using KAPA RNA HyperPrep Kit with RiboErase (Kapa Biosystems Inc.). The cDNA libraries were sequenced using Illumina HiSeq machine for paired-end reads using GENEWIZ (South Plainfield, NJ) services, and sequencing analysis was performed on the GALAXY platform [39]. Raw sequencing reads were aligned to the human genome assembly hg38 and mouse genome mm10, and reads were counted and annotated using featureCounts using GENCODE GRCh38 and GRCm38 comprehensive gene annotation. DESeq2 was used to normalize counts and determine differentially expressed genes (DEGs) using statistical analysis. Ontology enrichment analysis of DEGs was performed using PANTHER and Enrichr software [40, 41].

#### Statistical analysis

Data are presented as the mean ± standard error of the mean (SEM) of triplicates per analysis. Results with **p ≤ 0.01 were considered statistically significant. All analyses were performed using SPSS version 26 (SPSS Inc. USA) using the one‐way ANOVA test.

## Results

### Differentiation and characterization of pMSCs into RPCs

**Fig. 1:**
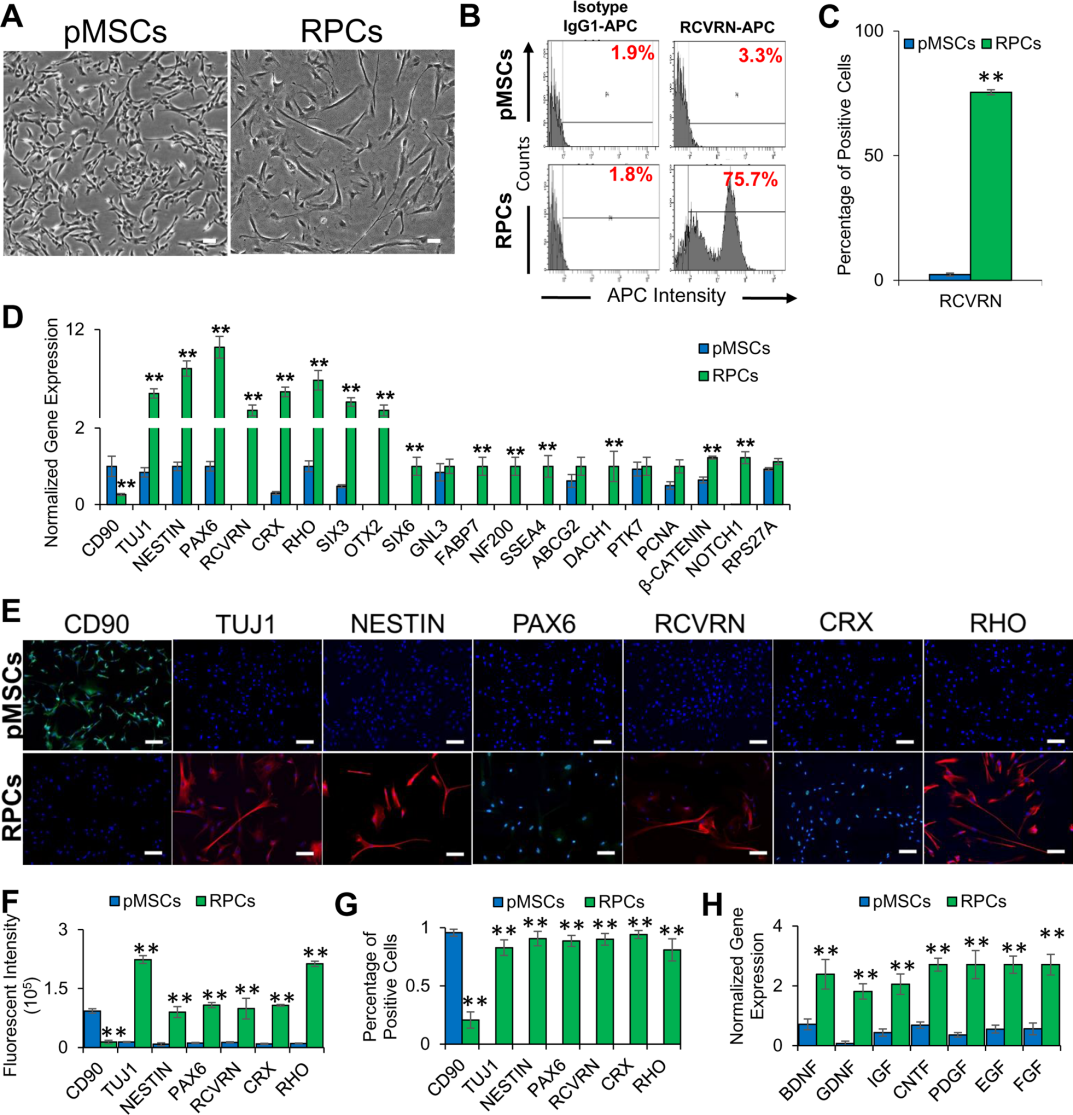
Characterization of RPCs derived from pMSCs. **A** Phase-contrast images of pMSCs and RPCs. 100 µm scale bar. (Magnification: ×4). **B**, **C** Histograms and graphical representation of the expression of the retinal marker, RCVRN, by pMSCs and RPCs as determined by flow cytometry (**p ≤ 0.01). **D** Expression of MSC (*CD90*), neural (*TUJ1, NESTIN,* and *PAX6*), retinal (*RCVRN, CRX, RHO, SIX3, OTX2, SIX6, GNL3, FABP7, NF200, SSEA4, ABCG2, DACH1, PTK7, PCNA, β-CATENIN, NOTCH1,* and *RPS27A*) genes in pMSCs and RPCs as determined by qRT-PCR. Gene expression was normalized to *GAPDH* and *β-ACTIN*. Error bars represent the SEM (**p ≤ 0.01). **E** Expression of MSC (CD90), neural (TUJ1, NESTIN, and PAX6), and retinal (RCVRN, CRX, and RHO) proteins in pMSCs and RPCs as visualized by immunocytostaining. Shown are representative merged images of DAPI (blue) and human antibodies (green and red). 100 µm scale bar (Magnification: ×10). **F** Measurement of fluorescent intensity of proteins immunocytostained using antibodies in pMSCs and RPCs (**p ≤ 0.01). **G** Percentage of positive immunocytostained cells (**p ≤ 0.01). **H** Expression of neurotrophic genes, *BDNF, GDNF, IGF, CNTF, PDGF, EGF,* and *FGF* in pMSCs and RPCs. Gene expression was normalized to *GAPDH* and *β-ACTIN*, and error bars represent the SEM (**p ≤ 0.01). Significant changes in the gene and protein expression were observed upon differentiation of pMSCs into RPCs. All experiments were carried out in triplicates

Previously isolated and characterized pMSCs [36] were differentiated into RPCs using a differentiation medium. The results showed that the differentiated cells exhibited neural outgrowth (Fig. 1A) and expressed RCVRN (Fig. 1B-C), a retinal marker, based on microscopic and flow cytometry analyses, respectively. They also expressed neural genes, *TUJ1, NESTIN* and *PAX6,* and retinal genes, *RCVRN, CRX, RHO, SIX3*, *OTX2, SIX6, GNL3, FABP7, NF200, SSEA4, ABCG2, DACH1, PTK7, PCNA, β-CATENIN, NOTCH1,* and *RPS27A* (Fig. 1D). The expression of neural and retinal markers was further validated by immunofluorescent staining using specific antibodies showed that pMSC-derived RPCs expressed neural (TUJ1 and NESTIN), and retinal (PAX6, RCVRN, CRX, and RHO) proteins (Fig. 1E–G). The estimated rate of differentiation of pMSCs into RPCs was 83%, 91%, 89%, 90%, 94%, and 81% based on the expression of TUJ1, NESTIN, PAX6, RCVRN, CRX, and RHO, respectively. RPCs also expressed high levels of neurotrophic factors, *BDNF, GDNF, IGF, CNTF, PDGF, EGF,* and *FGF* compared to pMSCs (Fig. 1H), suggesting that they could potentially promote neuroprotection.

### Transcriptomic analysis of pMSCs and RPCs

**Fig. 2:**
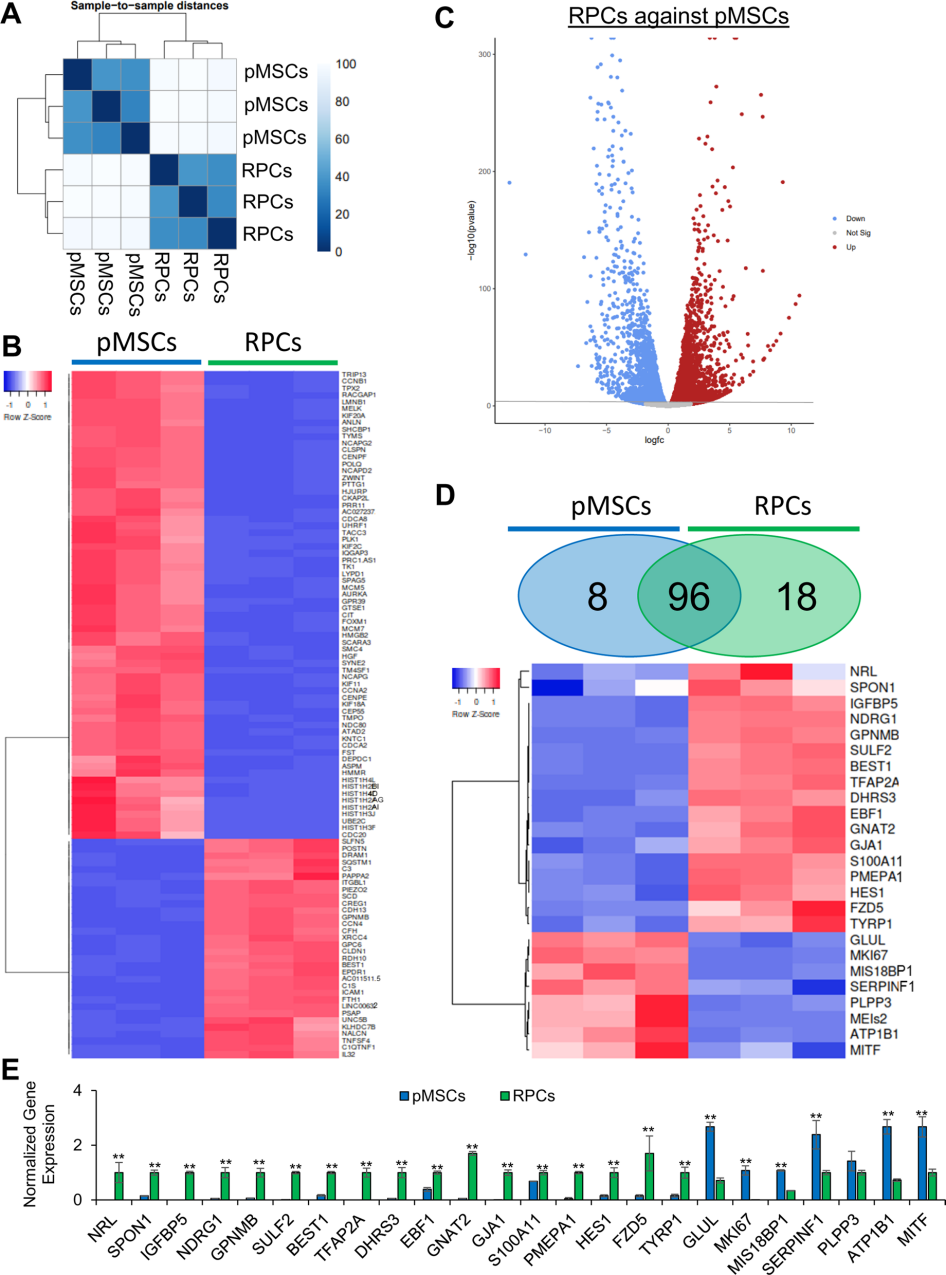
Transcriptome analysis of pMSCs and RPCs. **A** Hierarchical clustering analysis was performed using DESeq2, and the data were plotted as a sample-to-sample heatmap. The colored indicator represents sample distances (blue signifies a high correlation). **B** Heatmap showing raw z-scores of RNA-seq log2 transformed values of the top 100 DEGs and **C** volcano plot to visualize DEGs. **D** Heatmap showing raw z-scores of RNA-seq log2 transformed values of expression of retinal markers in pMSCs and RPCs. Venn diagrams depict the total number of genes upregulated in pMSCs (blue) and RPCs (green). In the heatmap, upregulated and downregulated genes are shown in red and blue, respectively. **E** Validation of selected retinal DEGs in pMSCs and RPCs by qRT-PCR. Gene expression was normalized to GAPDH and β-ACTIN, and error bars represent the SEM (**p ≤ 0.01). All experiments were performed in triplicate

To further characterize the RPCs, RNA-seq analysis was performed. The results depicted in Fig. 2A show a distinct difference in gene expression patterns between pMSCs and RPCs. This analysis revealed 2951 DEGs at a false discovery rate (FDR) < 0.05. Of these DEGs, 1,618 genes were upregulated in pMSCs, and 1,333 genes were upregulated in RPCs. Among the top 100 DEGs (FDR < 8.3 × 10^–147^), 68 and 32 genes were upregulated in pMSCs and RPCs, respectively (Fig. 2B, C). Notably, 8 upregulated DEGs in pMSCs were specific to RPE while 18 upregulated DEGs in RPCs were specific to the neuronal layers of the retina (FDR < 0.05) (Fig. 2D). The expression of selected DEGs was validated by qRT-PCR. The selected DEGs were associated with retinal cells including, photoreceptors *(SULF2, GNAT2 and NRL),* amacrine *(TFAP2A and ATP1B1),* horizontal *(NDRG1),* Müller glia *(HES1, and SPON1),* and ganglion *(EBF1)* cells (Fig. 2E).

**Fig. 3:**
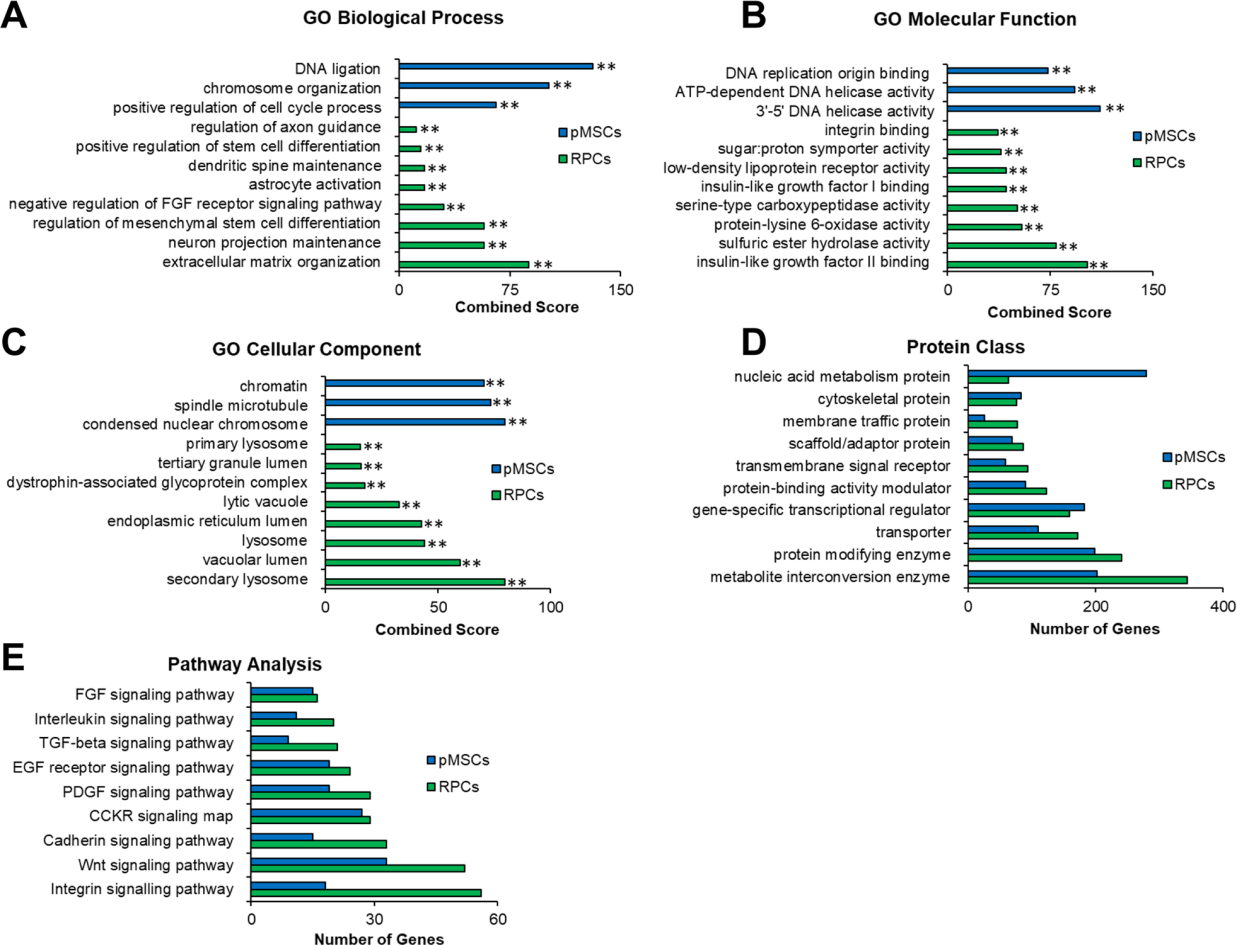
GO analysis of RNASeq of pMSCs and RPCs. Enrichr and PANTHER analyses of DEGs was performed with Benjamini–Hochberg FDR < 0.05 using DESeq2. **A**–**C** GO term enrichment analysis depicting upregulated DEGs associated with biological processes, molecular function, and cellular component, respectively, and the x-axis represents the combined score generated by Enrichr (**p ≤ 0.01). (D and E) Protein classes and pathways upregulated in pMSCs and RPCs, as determined by PANTHER analysis. The x-axis represents the number of genes associated with each category

We next performed functional analysis of the DEGs using gene ontology (GO) analysis. Comparative enrichment analysis of pMSCs and RPCs performed using Enrichr identified DEGs associated with biological process, molecular function, and cellular component ontologies. In the category of biological processes, pMSCs were highly enriched in DNA ligation, chromosome organization, and positive regulation of cell cycle process (Fig. 3A), while RPCs were significantly enriched in regulation of axon guidance, positive regulation of stem cell differentiation, dendritic spine maintenance, astrocyte activation, negative regulation of FGF receptor signaling pathway, and regulation of regulation mesenchymal stem cell differentiation, neuron projection maintenance, and extracellular matrix organization. Next, we investigated the category of molecular function (Fig. 3B). pMSCs were enriched in DNA replication origin binding, ATP-dependent DNA helicase activity, and 3'-5' DNA helicase activity, whereas RPCs presented a significant enrichment of integrin binding, sugar:proton symporter activity, low-density lipoprotein receptor activity, insulin-like growth factor I binding, serine-type carboxypeptidase activity, protein-lysine 6-oxidase activity, sulfuric ester hydrolase activity, and insulin-like growth factor II binding. We then investigated cellular components enriched in the DEGs. GO analysis showed that chromatin, spindle microtubule, and condensed nuclear chromosome were enriched in pMSCs, while RPCs displayed significant enrichment of primary lysosome, tertiary granule lumen, dystrophin-associated glycoprotein complex, lytic vacuole, endoplasmic reticulum lumen, lysosome, vacuolar lumen, and secondary lysosome (Fig. 3C).

Next, we employed PANTHER analysis to investigate differentially expressed protein classes (Fig. 3D), which showed that cytoskeletal and gene-specific transcriptional regulation were enriched in pMSCs and RPCs. However, nucleic acid metabolism proteins were more enriched in pMSCs than RPCs. In contrast, metabolite interconversion enzyme, protein modifying enzyme, transporter, protein-binding activity modulator, transmembrane signal receptor, scaffold/adaptor protein, and membrane traffic protein classes were associated with more genes in RPCs. In signaling pathways, FGF, interleukin, TGF-beta, EGFR, PDGF, CCKR, Cadherin, Wnt, and Integrin were more expressed in RPCs than pMSCs (Fig. 3E). Altogether, these results showed that RPCs derived pMSCs expressed expected retinal specific genes. Interestingly, they also expressed neurotrophic genes involved in neuroprotection. Therefore, RPCs were an ideal choice to investigate their therapeutic potential.

### Improvement of retinal function in rd12 mice transplanted with cells

**Fig. 4:**
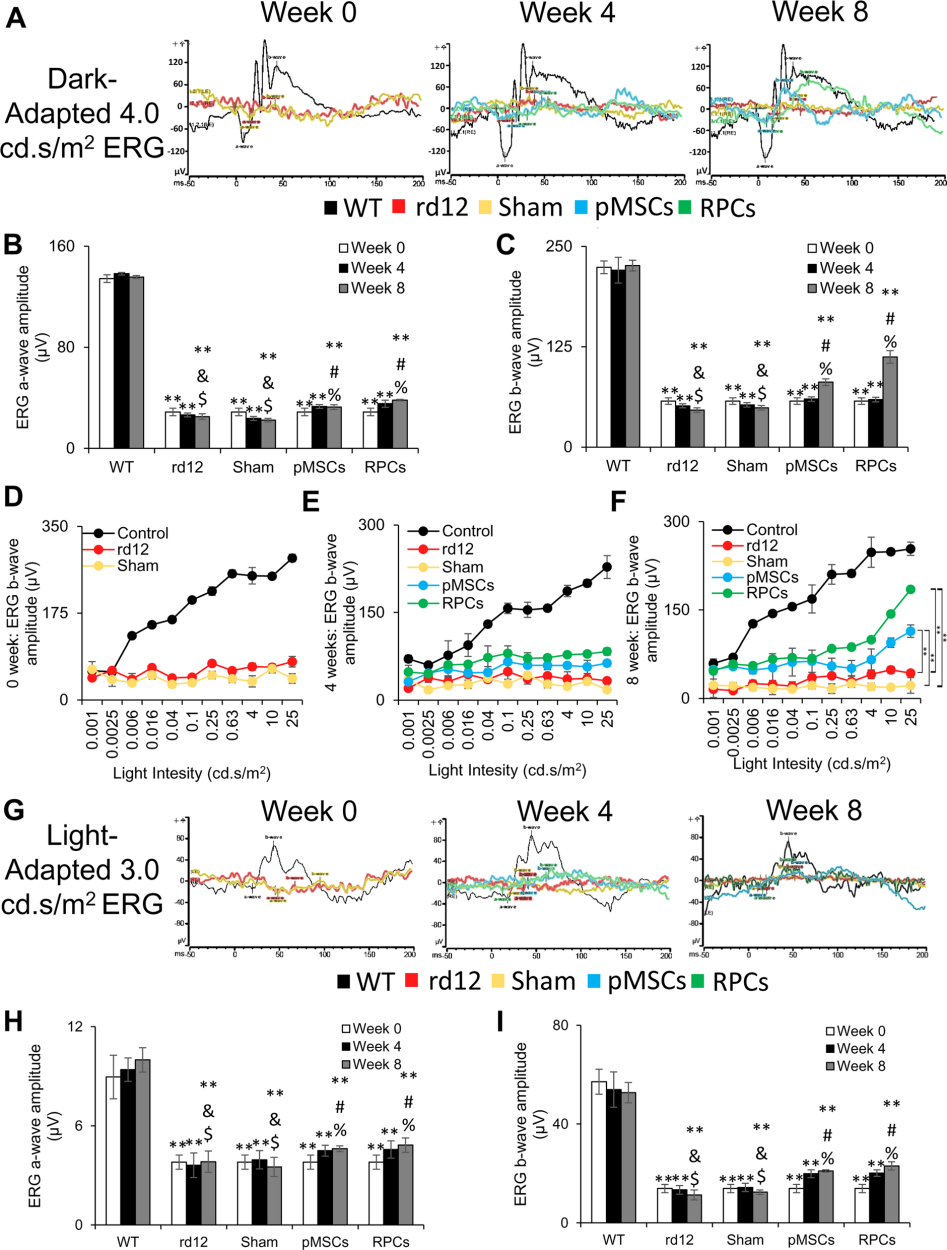
ERG analysis. **A** Representative dark-adapted ERG test of control (wild-type), rd12, sham, rd12 + pMSCs and rd12 + RPCs at 0, 4, and 8 weeks after transplantation of cells. **B**, **C** Graphical representation of the amplitude of the dark-adapted ERG a- and b-waves, respectively, from A. **D**–**F** Intensity-response curves of ERG b-waves at 0, 4, and 8 weeks, respectively, after transplantation of cells (**p < 0.01). **G** Representative light-adapted ERG test of control (wild-type), rd12, sham, rd12 + pMSCs and rd12 + RPCs at 0, 4, and 8 weeks after transplantation of cells. **H**, **I** Graphical representation of the amplitude of the light-adapted ERG a- and b-waves, respectively, from G. Symbols, **, #, %, & and $ indicate significant difference at *p* ≤ 0.01 between all experimental conditions: control (wild-type), rd12, sham, rd12 + pMSCs and rd12 + RPCs, respectively

The behavioral analysis of rd12 mice intravitreally transplanted with PKH26 labeled pMSCs and RPCs was performed for vision evaluation using ERG analysis. ERG analysis showed that dark- and light-adapted rd12 mice had almost a complete loss of a- and b-wave amplitudes (Fig. 4). A progressively significant increase in the a- and b-wave amplitudes of the dark-adapted animals was observed in pMSCs transplanted rd12 mice. Notably, an even greater increase in the a- and b-wave amplitudes was noticed in animals transplanted with RPCs than pMSCs (Fig. 4A–C). The increase in the amplitude was greater at 8 than 4 weeks after cell transplantation, indicating a progressive improvement in the vision of treated mice. In addition, the b-wave amplitude progressively improved with an increase in light intensity in rd12 mice treated with cells, but the increase was more pronounced with RPCs than pMSCs (Fig. 4D–F).

On the other hand, only the b-wave amplitude was progressively increased in the light-adapted rd12 mice transplanted with cells (Fig. 4G–I). Again, RPCs had a more significant effect in improving the b-wave amplitude than pMSCs, and the effect was more significant at 8 than 4 weeks after cell transplantation. Overall, these results showed greater improvement in retinal function by RPCs than pMSCs.

### Transplanted cells improved the retinal structure

**Fig. 5:**
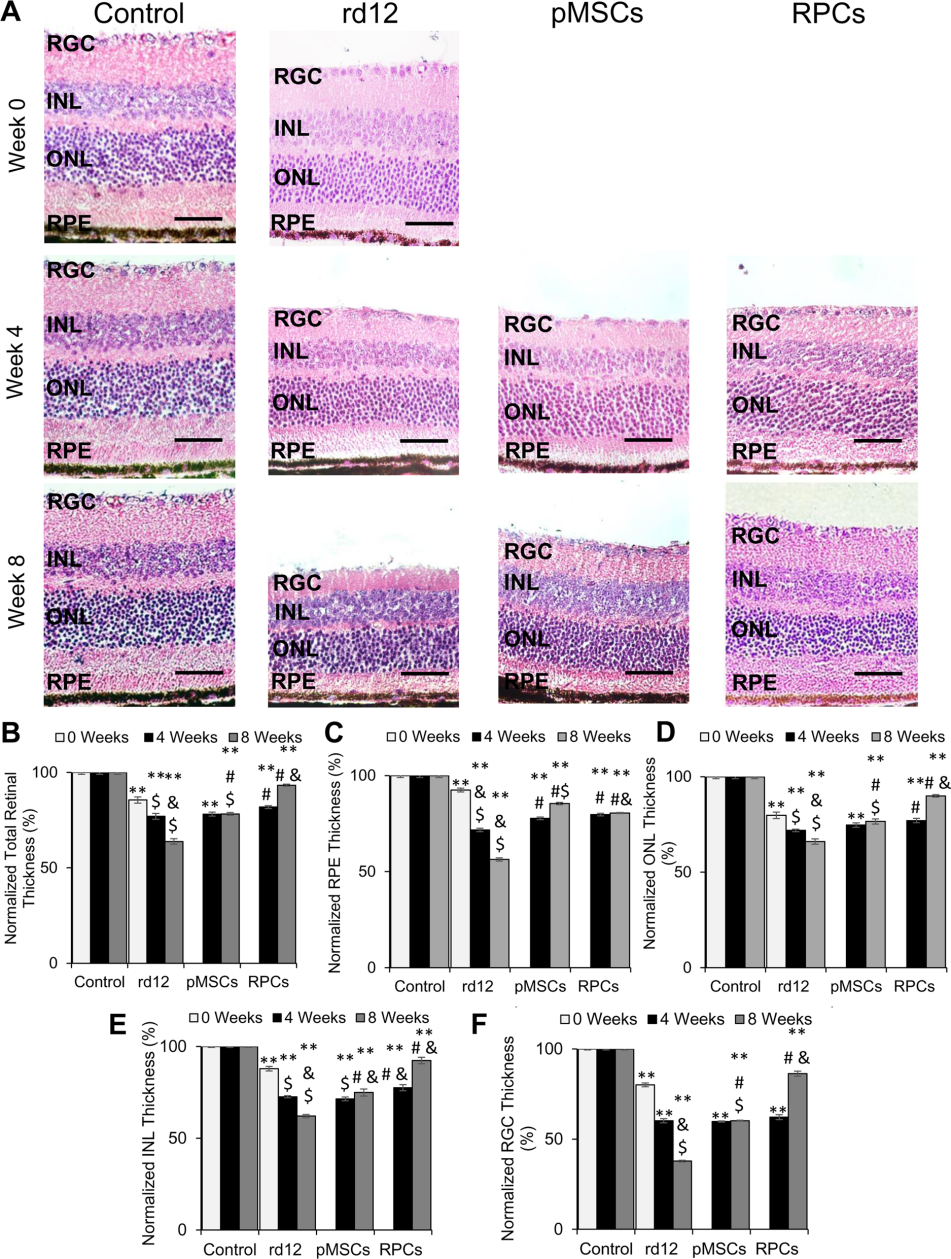
Histological analysis of rd12 retina transplanted with human cells. **A** H&E staining of paraffin-embedded sections of the control (wild-type), rd12, pMSCs and RPCs transplanted retina harvested at 0 week, 4 week, and 8 weeks. All scale bars represent 50 μm. (Magnification: ×40). **B**–**F** Graphical representation comparing the average thickness of the retina, RPE, ONL, INL, and RGC. Thickness of each retina was normalized to the control (wild-type) retina. Symbols, **, #, & and $ indicate significant difference at *p* ≤ 0.01 between all experimental conditions: control (wild-type), rd12, rd12 + pMSCs and rd12 + RPCs, respectively

We next analyzed the structure of the retina. The results depicted in Fig. 5 show that rd12 mice displayed a progressive decrease in thickness of the total retina, RPE, ONL, INL, and RGC during the 8-week study signifying degeneration of the retina (Fig. 5A–F). In rd12 mice transplanted with pMSCs, the degeneration of the retina was halted as there was no net decrease in the retinal thickness, except that there was a significant increase in the RPE layer after the 8-week period. After RPC transplantation, the thickness of the retina, ONL, INL, and RGC significantly increased (from 85.6 to 93.3%, 79.8–90.0%, 87.9–92.4%, and 80.1–86.3%, respectively). However, there was no change in the thickness of the RPE layer in RPC transplanted retina. The transplanted cells had no adverse effects as no tumor formation was observed. Overall, pMSCs halted the retinal regeneration while RPCs improved the retinal thickness.

### Transplanted cells homed to the retina

Cell tracking revealed that PKH26 labeled cells were localized in the retinal whole-mount of animals transplanted with cells (Additional file 3A). Differential interface contrast (DIC) microscopy confirmed the presence and dispersal of labeled cells in the retina (Additional file 3B, C). Overall, RPCs were more dispersed than pMSCs in the retina 8 weeks post-transplantation. These results suggest that transplanted cells homed to different retinal layers.

### Expression of human markers in the transplanted rd12 mice retina

**Fig. 6:**
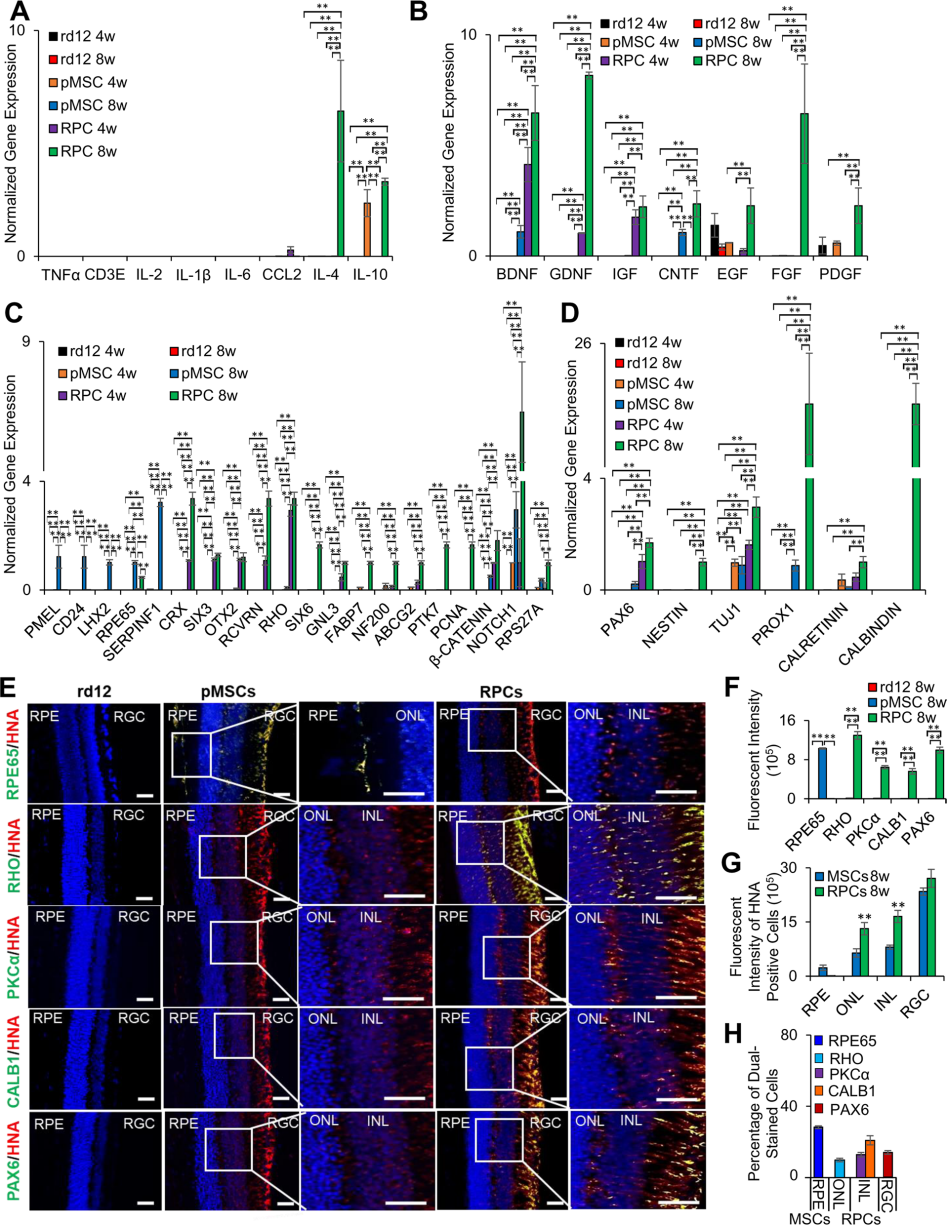
Post-transplantation analysis of the human markers in rd12 retina. **A** Expression of human pro- and anti-inflammatory genes, *TNFα, CD3E, IL-2, IL-1β, IL-6* and *CCL2, and IL-4* and *IL-10,* respectively*,* **B** neuroprotective genes, *BDNF, GDNF, IGF, CNTF, EGF, FGF, and PDGF,* **C** retinal genes, *PMEL, CD24, LHX2, RPE65, SERPINF1, CRX, SIX3, OTX2, RCVRN, RHO, SIX6, GNL3, FABP7, NF200, ABCG2, PTK7, PNCA, β-CATENIN, NOTCH1,* and *RPS27A.* **D** neurogenesis genes, *PAX6, NESTIN, TUJ1, PROX1, CALRETININ,* and *CALBINDIN*. All gene expression was normalized to *GAPDH* and *β-ACTIN*. Error bars represent the SEM of triplicate experiment (**p ≤ 0.01). **E** Immunohistochemical analysis of paraffin-embedded sections of the retina showing expression of human (RPE65, RHO, PKCα CALB1, and PAX6) proteins. The sections were countered stained with HNA (red). Blue and green colors represent the DAPI staining of the nuclei and human proteins, respectively. The right column shows enlarged merged images (over exposed) of only the area in the box to enhance the contrast. Scale bars represent 50 µm scale bars. (Magnification: ×40). **F** Measurement of fluorescent intensity of proteins as shown in E (**p ≤ 0.01). **G** Fluorescent intensity for the quantification of the number of HNA-positive cells in the RPE, ONL, INL and RGC layers of the retina (**p ≤ 0.01). **H** Percentage of dual-stained transplanted cells for HNA with RPE65, RHO, PKCα, CALB1, and PAX6 in each of the retina layers (**p ≤ 0.01)

Since we found that the transplanted cells were localized in different retinal layers, it was conceivable that they would express various retinal markers. The expression of human markers was first investigated by transcriptional analysis. The results showed the expression of human genes associated with anti-inflammation, neuroprotection, retinal, and neurogenesis. In general, fewer human genes were expressed in animals transplanted with pMSCs than RPCs. Among the anti-inflammatory genes, only expression of *IL-10* was observed 4 weeks after pMSC transplantation, but *IL-4* and *IL-10*, were expressed after 8 weeks in the case of RPCs (Fig. 6A). No pro-inflammatory human genes were expressed in either pMSC or RPC transplanted animals.

The expression of two neuroprotective genes, *BDNF* and *CNTF,* was detected only 8 weeks after transplantation of pMSCs. In RPCs, some neuroprotective genes (*BDNF*, *GDNF*, and *IGF*) were expressed at 4 weeks. However, several genes, *BDNF*, *GDNF*, *IGF*, *CNTF*, *EGF*, *FGF*, and *PDGF* were expressed 8 weeks after transplantation (Fig. 6B).

As for the retinal genes, pMSCs expressed human RPE genes, *PMEL, CD24, LHX2, RPE65*, and *SERPINF1* only at 8 weeks after transplantation, whereas RPC transplanted animals expressed human genes, *CRX, SIX3, OTX2, RCVRN*, *RHO, SIX6, GNL3, FABP7, NF200, ABCG2, PTK7, PCNA, β-CATENIN, NOTCH1, and RPS27A* (Fig. 6C), which are predominantly associated with the neural layers (ONL, INL, and RGC) of the retina. The expression of ONL, INL, and RGC genes suggests that transplanted RPCs successfully differentiated and integrated into the neural layers of the retina. In contrast, the expression of RPE genes suggests that pMSCs are differentiated and integrated into the RPE layer of the retina.

Only two neurogenesis genes, *TUJ1* and *PROX1*, were expressed at 8 weeks after transplantation of pMSCs, but several neurogenesis genes, *PAX6, NESTIN, TUJ1, PROX1, CALRETININ*, and *CALBINDIN*, were expressed at 8 weeks (with only *PAX6* and *TUJ1* expressed at 4 weeks) after transplantation of RPCs (Fig. 6D).

### Differentiation of cells in the transplanted retina

Immunofluorescent staining of the retinal sections with human specific antibodies revealed that transplanted cells migrated into various retinal layers as indicated by the human nuclear antigen (HNA) staining and some of these cells co-expressed retinal markers (RPE65, RHO, PKCα, CALB1, and PAX6) (Fig. 6E–H) [42–44]. Sign of migration of HNA-positive cells into RPE and ONL was first observed 4 weeks after transplantation of the retina with pMSCs and RPCs, respectively (data not shown). Quantification of the intensity of the immunostaining of the retina transplanted with pMSCs showed that 28% of HNA cells were also positive for RPE65, a marker of the RPE layer, whereas in the retina transplanted with RPCs, 10% of the HNA cells were positive for RHO (ONL), 13% and 21% of HNA cells were positive for PKCα and CALB1 (INL), respectively, and 10% of HNA cells were positive for PAX6 (RGC). Dual stained cells were dispersed and integrated into the various layers of the retina. Transplanted pMSCs were predominantly found in the RPE layer of the retina, suggesting that pMSCs differentiated towards the RPE lineage. In contrast, animals transplanted with RPCs showed a migration into almost all neural layers of the retina. They were detected in the ONL, INL, and RGC, suggesting that RPCs potentially differentiated into photoreceptors, amacrine, horizontal, bipolar, and ganglion cells.

### Transplanted cells promoted endogenous mouse gene expression

**Fig. 7:**
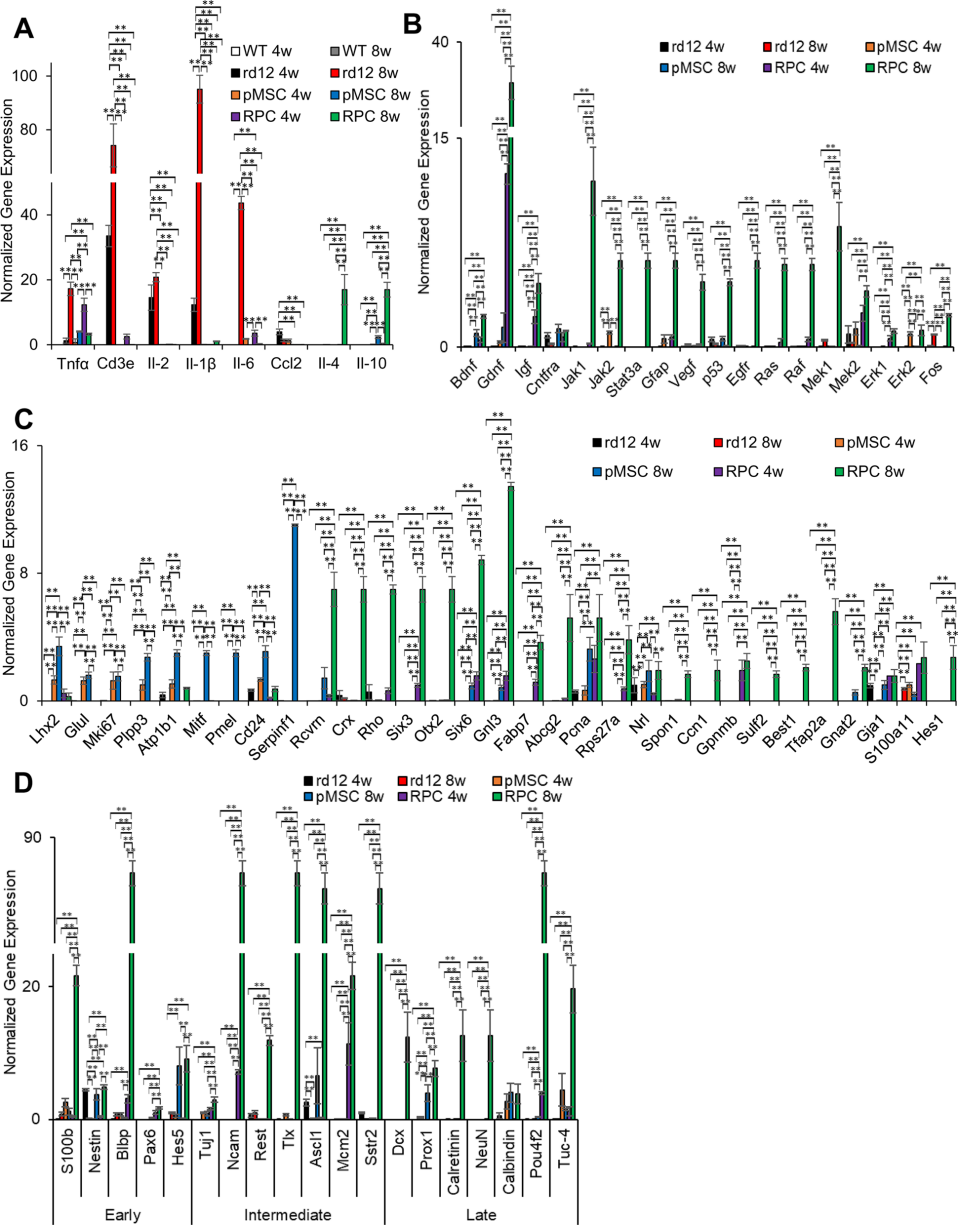
Effect of transplanted cells on the expression of endogenous mouse **genes in rd12 retina.** **A** Expression of pro-inflammatory genes (*Tnfα, Cd3e, Il-2, Il-1β, Il-6* and *Ccl2) and* anti-inflammatory genes (*Il-4* and *Il-10*), **B** neuroprotective genes *(Bdnf, Gdnf, Igf, Cntfra, Jak1, Jak2, Stat3a, Gfap, Vegf, p53, Egfr, Ras, Raf, Mek1, Mek2, Erk1, Erk2, and Fos*), **C** retinal genes (*Lhx2, Glul, Mki67, Plpp3, Atp1b1, Mitf, Pmel, Cd24, Serpinf1, Rcvrn, Crx, Rho, Six3, Otx2, Six6, Gnl3, Fabp7, Abcg2, Pcna, Rps27a, Nrl, Spon1, Ccn1, Gpnmb, Sulf2, Best1, Tfap2a, Gnat2, Gja1, S100Aa11,* and *Hes1) and* **D** genes involved in early (*S100b, Nestin, Blbp, Pax6,* and *Hes5*), intermediate (*Tuj1, Ncam, Rest, Tlx, Ascl1, Mcm2,* and *Sstr2*) and late (*Dcx, Prox1, Calretinin, NeuN, Calbindin, Pou4f2,* and *Tuc-4*) stage of neurogenesis. All gene expression was normalized to *Gapdh* and *β-Actin*, and error bars represent the SEM of the triplicate experiment (**p ≤ 0.01)

To evaluate the effect of the transplanted cells on the rd12 mouse retina, we investigated endogenous mouse genes associated with inflammation, neuroprotection, retina, and neurogenesis. It has been reported that pro-inflammatory genes are upregulated in rd12 mice [45]. Our study also found that *Tnfα, Cd3e, Il-2, Il-1β, Il-6,* and *Ccl2* were highly expressed in rd12 mice but not in the wild-type mice (Fig. 7A). However, the expression of these genes was reduced to near wild-type levels in transplanted animals. Interestingly, the expression of anti-inflammatory genes, *ll-4* and *Il-10,* increased in rd12 mice 8 weeks after transplantation of RPCs. Only *ll-10* was expressed at a much lower level in pMSCs than RPCs.

Among the neuroprotective genes, *Jak2*, *Erk2*, and *Mek2* as well as *Bdnf* and *Cntfra*, were upregulated in the retina of rd12 mice 4 and 8 weeks, respectively, after pMSC transplantation (Fig. 7B), whereas *Gdnf*, *Igf*, and *Mek2* and *Bdnf, Gdnf, Igf, Jak1, Jak2, Stat3a, Gfap, Vegf, p53, Egfr, Ras, Raf Mek1, Mek2, Erk1, Erk2,*, and *Fos*, were upregulated in the retina of animals 4 and 8 weeks, respectively, after transplantation of RPCs (Fig. 7B).

In addition, several RPE genes, *Lhx2, Glul, Mki67, Plpp3, Atp1b1, Cd24* and *Serpinf1* were upregulated in the rd12 mice retina 4 weeks after transplantation of pMSCs, and their expression was further increased by 8 weeks. In addition, *Mitf and Pmel* were expressed 8 weeks after transplantation of pMSCs. In the case of RPCs, noticeable expression of *Six3*, *Six6, Gnl3, Fabp7, Pcna, Rps27A, Gpnmb, Gja1, and S100a11* was observed at 4 weeks which further increased by 8 weeks along with expression of several other retinal genes, *Crx, Otx2, Rcvrn*, *Rho, Abcg2, Nrl, Spon1, Ccn1, Sulf2, Best1, Tfap2a,* and *Hes1* (Fig. 7C).

We also saw the upregulation of neurogenesis genes, which are involved in the retina's early, intermediate, and late stages. For example, the expression of *S100b, Calbindin, and Tuc-4* (after 4 weeks) and *Nestin, Hes5, Ascl1,* and *Prox1* (after 8 weeks) was increased in animals transplanted with pMSCs, whereas the expression of *Ncam*, *Mcm2*, and *Pou4f2* (after 4 weeks) and *S100b, Nestin, Blbp, Pax6*, *Hes5, Tuj1, Ncam, Rest, Tlx, Ascl1, Mcm2, Sstr2*, *Dcx, Prox1, Calretinin, NeuN, Pou4f2*, and *Tuc-4* (after 8 weeks) was increased in animals transplanted with RPCs (Fig. 7D). Taken together, RPCs were more effective in upregulating the mouse genes involved in inflammation, neuroprotection, retina, and neurogenesis.

### Effect of transplanted cells on the transcriptome of rd12 mouse retina

**Fig. 8:**
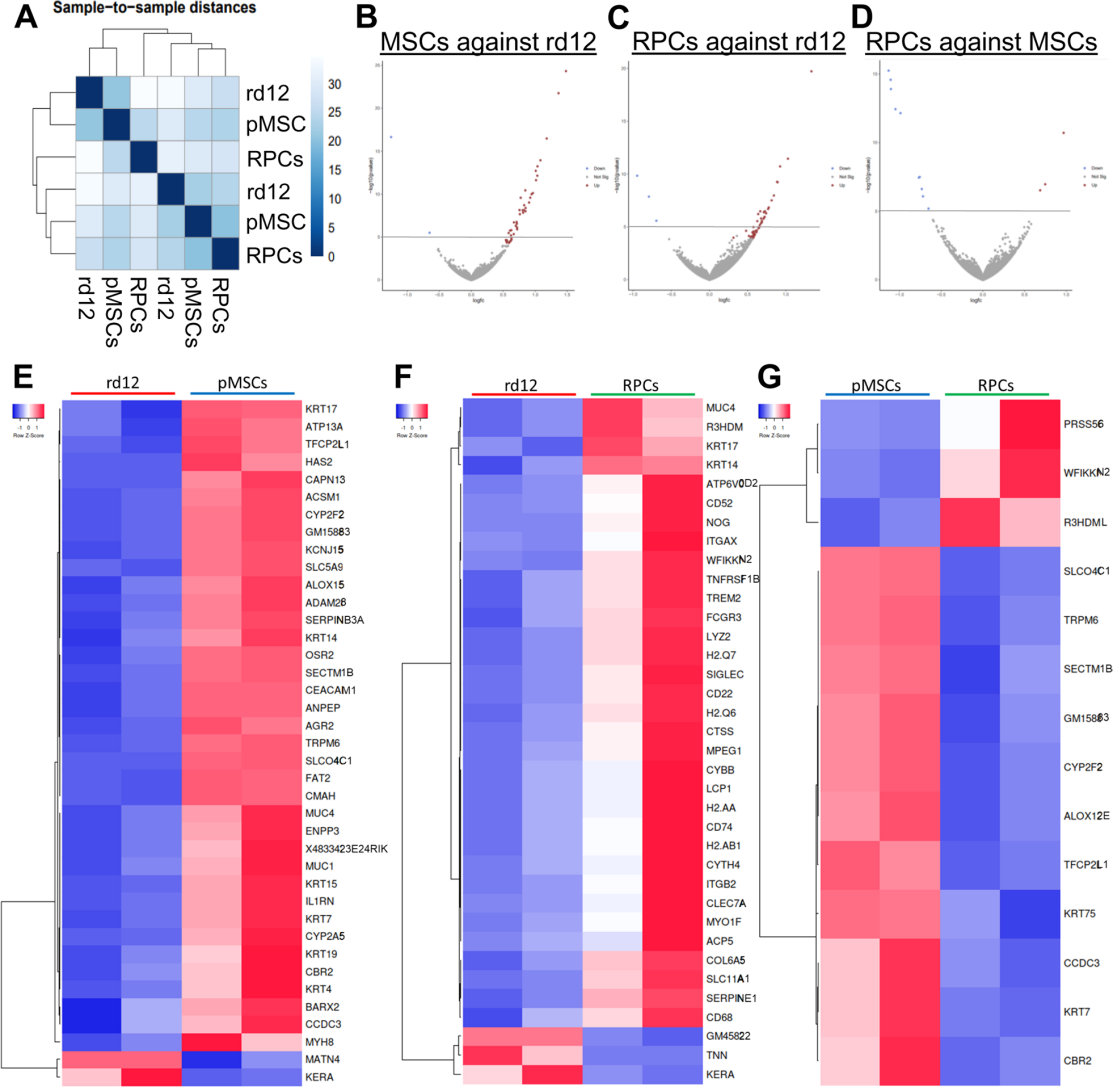
Transcriptome analysis of rd12 mice retina transplanted with cells*.* **A** RNA-seq data were analyzed to measure sample distances to evaluate for similarities. Hierarchical clustering was performed using DESeq2, and the data were plotted as a sample-to-sample heatmap. The colored indicator represents sample distances (blue signifies a high correlation). (B-G) Heatmap and volcano plots showing raw z-scores of RNA-seq log2 transformed values of the top DEGs in retina of rd12 vs rd12 + pMSCs (**B**, **E**), rd12 vs rd12 + RPCs (**C**, **F**), and rd12 + pMSCs vs rd12 + RPCs (**D**, **G**). Upregulated and downregulated genes are red and blue, respectively

RNA-seq analysis depicted in Fig. 8A shows hierarchical clustering of rd12 mice vs. rd12 mice transplanted with cells. Expectedly, the untreated rd12 mice formed a clear cluster. In the case of the transplanted animal groups, pMSCs and RPCs showed a higher gene correlation than the untreated animals. Figure 8B, E identifies 39 DEGs (37 upregulated and 2 downregulated genes) at *p* value < 0.05 in rd12 mice vs animals transplanted with pMSCs, whereas 36 DEGs (33 upregulated and 3 downregulated genes) at *p* value < 0.05 were found in rd12 mice vs animals transplanted with RPCs (Fig. 8C, F). The comparison between rd12 mice transplanted with pMSCs and RPCs revealed 14 DEGs (11 and 3 upregulated genes in pMSCs and RPCs, respectively) at *p* value < 0.05 (Fig. 8D, G).

**Fig. 9:**
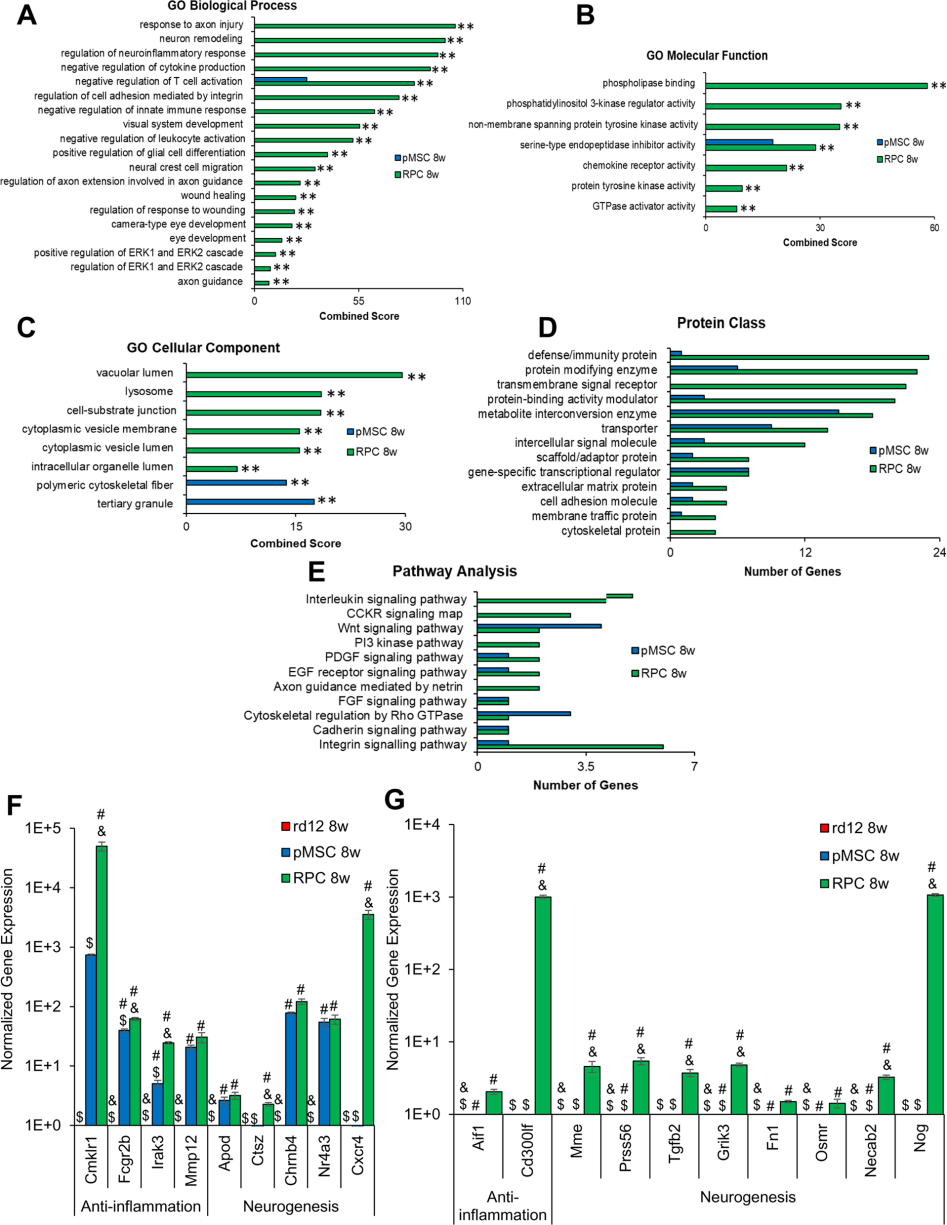
GO analysis of top mouse DEGs in rd12 mice retina transplanted with cells and validation by qRT-PCR. Enrichr and PANTHER analyses of DEGs were performed with *p* < 0.05 using DESeq2 after 8 week cell transplantation. **A**–**C** GO term enrichment analysis of upregulated DEGs associated with biological processes, molecular function, and cellular component, respectively, and the x-axis represents the combined score generated by Enrichr (**p ≤ 0.01). **D**, **E** Protein classes and pathways upregulated in rd12 + pMSCs and rd12 + RPCs, as determined by PANTHER analysis. The x-axis represents the number of genes associated with each category. **F**, **G** Expression of anti-inflammatory and neurogenesis genes in rd12, rd12 + pMSCs and rd12 + RPCs and expression ± SEM normalized to rd12 and r12 + pMSCs, respectively, as determined by qRT–PCR. Symbols, #, & and $ indicate significant difference at *p* ≤ 0.01 between all experimental conditions: rd12, rd12 + pMSCs and rd12 + RPCs, respectively

Further analysis of the DEGs, *p* value < 0.05 using GO terms identified several GO categories enriched in transplanted animals. In the biological process category, only negative regulation of T cell activation was enriched in rd12 mice transplanted with pMSCs (Fig. 9A). However, in RPC transplanted animals, several processes including response to axon injury, neuron remodeling, regulation of neuroinflammatory response, negative regulation of cytokine production, negative regulation of T cell activation, regulation of cell adhesion mediated by integrin, negative regulation of innate immune response, visual system development, negative regulation of leukocyte activation, positive regulation of glial cell differentiation, neural crest cell migration, regulation of axon extension involved in axon guidance, wound healing, regulation of response to wounding, camera-type eye development, eye development, positive regulation of ERK1 and ERK2 cascade, regulation of ERK1 and ERK2 cascade, and axon guidance were enriched.

In the molecular function category, again, only serine-type endopeptidase inhibitor activity was enriched in rd12 mice transplanted with pMSCs. But several molecular functions including serine-type endopeptidase inhibitor activity, phospholipase binding, phosphatidylinositol 3-kinase regulator activity, non-member spanning protein tyrosine kinase activity, chemokine receptor activity, protein tyrosine kinase activity, and GTPase activator activity were enriched in rd12 mice transplanted with RPCs (Fig. 9B).

In the cellular component category, polymeric cytoskeletal fiber and tertiary granule were enriched in rd12 mice transplanted with pMSCs, whereas RPCs treated animals were enriched in several cellular components, including vacuolar lumen, lysosome, cell-substrate junction, cytoplasmic vesicle membrane, cytoplasmic vesicle lumen, and intracellular organelle lumen (Fig. 9C).

DEGs analyzed by PANTHER revealed that protein classes, metabolite interconversion enzyme, transporter, gene-specific transcriptional regulator, extracellular matrix protein, cell adhesion molecule, and membrane traffic protein were enriched in mice transplanted with pMSCs and RPCs. However, RPC transplanted animals had additional enriched protein classes including defense/immunity protein, protein modifying enzyme, transmembrane signal receptor, protein-binding activity modulator, intercellular signal molecule, scaffold/adaptor protein, and cytoskeleton protein (Fig. 9D).

For pathway analysis, we found that PDGF, EGF receptor, FGF, and cadherin signaling were enriched in rd12 transplanted pMSCs and RPCs. However, Wnt signaling and cytoskeletal regulation by rho GTPase pathways were also enriched in pMSCs, while interleukin, CCKR, PI3 kinase, axon guidance mediated by netrin and integrin signaling pathways were only enriched in the case of RPCs (Fig. 9E).

DEGs were further validated by quantitative gene expression analysis. The results depicted in Fig. 9F, G show that anti-inflammation and neurogenesis genes were significantly upregulated by several log-folds in transplanted animals compared to the rd12 mice. In general, anti-inflammation genes, *Cmklr1, Fcgr2b Irak3*, and *Cd300lf,* and neurogenesis genes, *Ctsz, Cxcr4, Mme, Prss56, Tgfb2, Grik3, Necab2,* and *Nog,* were significantly more upregulated in rd12 mice transplanted with RPCs than pMSCs. These results suggest that pMSCs induced genes involved immune response and RPE regeneration, whereas RPCs promoted expression of genes associated with immune response, neuroprotection, various neural layers of the retina, and neurogenesis.

## Discussion

This study differentiated pMSCs into RPCs to treat retinal degeneration in the rd12 mice, which improved retinal function. Before transplantation, RPCs were characterized for morphological and biochemical properties. pMSC-derived RPCs displayed neural extensions and expressed neural and retinal specific markers similar to published reports [46–48]. Transcriptomic analysis revealed that while genes associated with cell growth and proliferation (i.e., *CCNB1, CDC20,* and *CENPF* [49]) were highly expressed in pMSCs, they were significantly downregulated in RPCs. In addition, a number of retinal specific genes [50], *GPNMB, BEST1,* and *TYRP1* (RPE), *TFAP2A* and *ATP1B1* (amacrine), *NDRG1* (horizontal), *HES1* and *SPON1* (Müller glia), *SULF2, GNAT2,* and *NRL* (photoreceptor), and *EBF1* (RGC) were upregulated in RPCs. GO analysis of the DEGs indicated that RPCs were enriched in biological processes associated with neuron projection and dendritic spine maintenance involved in neural differentiation [51].

Following cell transplantation, a quantitative analysis of the retinal function was performed by ERG. As expected, both a- and b-waves amplitudes were diminished in untreated animals [52]. However, there was a significant improvement in the a- and b-wave amplitudes in the dark-adapted rd12 mice transplanted with cells. Notably, a greater improvement was observed in a- and b-wave amplitudes in animals treated with RPCs than pMSCs. In a previous study, a- and b-wave amplitudes were higher in dark-adapted RCS rats when treated with hBM-MSCs than untreated animals. However, the amplitudes continued to decrease over time [15]. Another study reported a combination of hBM-MSCs and fetal RPCs was used to treat retinal degeneration and showed an increase in the a- and b-wave amplitudes of dark-adapted RCS rats compared to untreated animals [17]. Again, the amplitudes progressively deteriorated even after cell transplantation. In contrast, subretinal injection of RPE-like cells derived from ESCs into rd12 mice displayed an increase in b-wave amplitude over the untreated animals but demonstrated a diminishing effect over time [53]. Feline Müller cells injected into the vitreous of a feline ganglion cell depletion model improved retinal function [54]. Unlike these reports, our results for the first time demonstrated a temporal improvement in the a- and b-wave amplitudes, particularly in the case of RPCs, suggesting a lasting effect of cell transplantation.

Post-transplantation histological analysis revealed temporal degeneration of retina in rd12 mice as previously described [55]. As expected, the retinal thickness in rd12 mice was decreased compared to the wild-type animals. In contrast, retinal thickness was improved in transplanted animals. RPCs had more significant improvement than pMSCs. These results suggest that while pMSCs provided protection that led to retinal thickness maintenance, RPCs halted degeneration and promoted retinal regeneration. In general, the thickness of all three nuclear layers, ONL, INL, and RGC, was significantly and progressively increased in the cell transplanted animals compared with rd12 mice. However, more improvement was observed in the ONL, INL, and RGC in RPC transplanted mice. In reported studies, transplanted MSCs have improved either ONL or RGC but not both [12, 56]. Therefore, our results are significant and exciting as they exhibited improvement in almost all nuclear layers, particularly in RPCs.

Previously, ESC retinal derivatives were found to survive and migrate between the ONL and RPE layers upon subretinal transplantation [53], whereas fetal RPCs were found to migrate towards the INL and ONL [33]. However, our cell tracking analysis showed localization of labeled cells at various sites of the retinal whole-mount, suggesting that transplanted cells dispersed from the injection site. DIC analysis also indicated the localization of labeled cells in multiple layers of the retina.

Since MSCs are known to exert paracrine effects [57], we investigated the expression of human inflammatory cytokines and neurotrophic factors in the retina of rd12 mice. Our results indicated high levels of xenogenic expression of human anti-inflammatory and neuroprotective genes in animals treated with pMSCs and RPCs. No pro-inflammatory markers were expressed, suggesting that transplanted cells did not exert an immune response and were safe for cell therapy. Several human retinal and neurogenesis genes were expressed in the transplanted animals, presumably providing neural protection and regeneration. Supporting these findings, xenogeneic protein expression of retinal markers was also observed in the retina of transplanted animals. In general, xenogeneic expression was more significant in RPC than in pMSC transplanted animals. Although several reports studied the effectiveness of cells in treating RDD in animal models [15, 56, 58], xenogenic expression of neuroprotective and neurogenesis markers has not been well investigated. It is conceivable that retinogenesis may in part be due to the differentiation of transplanted cells.

In retinal degeneration, expression of pro-inflammatory genes has been reported to increase with a subsequent decrease in the expression of retinal genes and vision loss [11, 13, 59]. We also found upregulation of several mouse pro-inflammatory genes, *Tnfα, Cd3e, Il-2, Il-1b, Il-6,* and *Ccl2*, and downregulation of several retinal genes in rd12 mice. Therefore, we investigated the effect of transplanted cells on the expression of inflammatory and retinal genes. Our study showed that transplanted cells induced endogenous inflammatory response by suppressing pro-inflammatory genes while activating anti-inflammatory and retinal genes. RPCs were more potent than pMSCs in exerting these beneficial effects. We also found that the transplanted cells, specifically RPCs, significantly upregulated several endogenous neurotrophic factors important for cell proliferation, survival, and neuronal differentiation [60, 61]. This observation supports the improvements in the structure and function recorded by H&E and ERG analyses, respectively.

In contrast to birds, amphibians, and fish, de novo neurogenesis and regenerative capacity of the adult mammalian retina is very limited [62, 63]. Therefore, only a few studies have investigated neurogenesis resulting from the augmentation of cells into the mammalian retina [9, 64]. We observed that mouse RPE and neural gene expression were increased in animals transplanted with pMSCs and RPCs, respectively. In addition, we investigated neurogenesis genes in the retina of animals transplanted with cells. The expression of only a few genes, *Nestin, Hes5,* and *Prox1,* increased 8 weeks after transplantation of pMSCs, whereas the expression of several genes involved in early, intermediate, and late neurogenesis was significantly increased in animals transplanted with RPCs, and their expression was even greater 8 weeks after transplantation. Altogether, our results suggest that pMSCs may be involved in the regeneration of mouse RPE, whereas RPCs promoted regeneration of the ONL, INL, and RGC. Nevertheless, further studies would be helpful to determine the specific cell types involved in the regeneration of rd12 mice retina.

Therefore, we investigated whether transplanted cells differentiated into the retinal lineage. Our results showed that transplanted pMSCs differentiated and integrated into the RPE layer as indicated by the dual expression of RPE65 and HNA. HNA-positive cells in the pMSC transplanted retina were not positive for any of the specific neural layer markers. On the other hand, transplanted RPCs appeared to differentiate and integrate into various neural layers where they expressed human-specific markers for RHO (rod), PKCα (rod bipolar), CALB1 (horizontal), and PAX6 9 (RGC) [42–44]. Although RHO was highly expressed in RPC transplanted retina, only about 10% of the HNA-positive cells were localized in the ONL. Our results showed that a significant number of RPCs also differentiated and migrated to other neural layers as judged by the dual staining of HNA with ONL, INL, and RGC specific markers.

The transcriptomic analysis also revealed significant differences in the animals treated with cells compared to rd12 mice. Analysis of RNA-seq data showed that large dispersions in the sample groups did not affect differential gene expression. We found a clear and significant difference in the DEGs by analyzing the gene expression pattern affected by pMSCs and RPCs. Comparative heatmap and volcano plot analyses revealed differences between animals transplanted with pMSCs and RPCs only in a select number of DEGs. GO analysis indicated several biological processes enriched in the transplanted animals. In the case of pMSCs, DEGs were involved in the suppression of T cell activation, whereas RPCs not only suppressed the expression of genes involved in the immune response but also promoted the enrichment of biological processes associated with neuron remodeling, visual system, and eye development, as well as axon guidance. This pattern of enrichment suggested that the transplantation of pMSCs and RPCs were effective in promoting anti-inflammatory response. RPCs promoted retinal regeneration, which led to greater improvement in visual function. These findings also complement the ERG results.

When DEGs were subjected to PANTHER analysis, activation of several pathways in the transplanted animals was revealed. Most of the signaling pathways involved in neural development were activated in both pMSCs and RPCs, except that interleukin, CCKR, PI3k, and axon guidance signaling pathways were activated only in RPCs. qRT-PCR analysis of DEGs validated the activation of genes involved in anti-inflammation and neurogenesis, which were expressed at higher levels in RPCs than pMSCs. Since qRT-PCR is a highly sensitive technique, the differences in DEG expression were many log folds higher. Some highly expressed genes in RPC transplanted animals were also associated with anti-inflammatory response [65], while the other highly expressed genes are known to play a role in neuronal and retinal development. For example, *Necab2* and *Prss56* are associated with neuronal development and ocular axial growth, respectively [66, 67]. Another gene, *Grik2*, acts as an excitatory neurotransmitter [68]. Altogether, these results demonstrated that RPCs countered inflammation and induced neuroprotection and neurogenesis that improved retinal structure and physiological function.

**Fig. 10:**
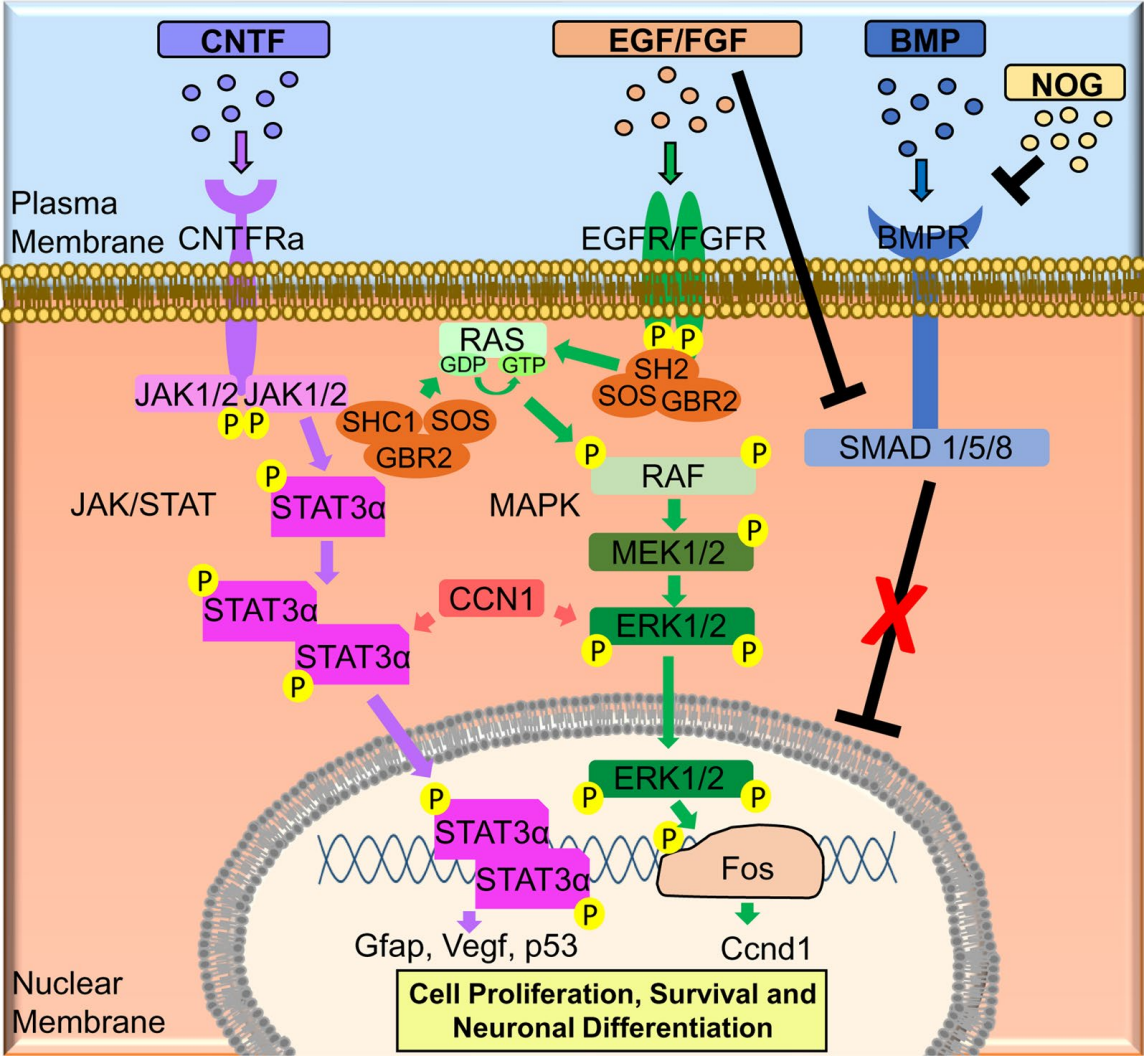
Proposed molecular mechanism involved the neuroprotection and retinal regeneration in rd12 mice transplanted with RPCs

The molecular analysis of RPC transplanted animals led us to propose a mechanism for improving retinal protection and regeneration (Fig. 10). In this mechanism, upregulation of CNTF could activate the JAK/STAT pathway. RPC transplanted animals expressed high levels of CCN1 that can also activate this pathway. In addition, activated STAT3α can turn on the genes (i.e., *Gfap, Vegf* and *p53*) involved in cell proliferation and survival. In addition, EGF/FGF may activate MAPK signaling pathways, which turn on the *Ccnd1* gene, known to be involved in proliferation and neuronal differentiation. Lastly, NOG will bind to the BMPR to inhibit the BMP pathway, which can also be inhibited by FGF signaling.

## Conclusion

In summary, our study demonstrated that RPCs counter inflammation, provided retinal protection, and promoted neurogenesis, resulting in improved retinal structure and physiological function in rd12 mice. Although several small clinical trials have utilized hBM-MSCs and hUC-MSCs, the results are not always conclusive [27, 69, 70]. Our investigation provided proof of concept and basis for large-scale preclinical and clinical studies to treat RDD using RPCs.

## Supplementary Information

**Additional file 1**. List of human primer sequences used in qRT-PCR

**Additional file 2.** List of mouse primer sequences used in qRT-PCR

**Additional file 3.** Tracking of cells transplanted into rd12 retina. (A) Whole-mount retina stained with RCVRN (green) 8 weeks after transplantation of PKH26-labeled (red) pMSCs and RPCs. 500 µm scale bar and 20 µm scale bars (inserts). (Magnification: ×5 and ×40, respectively). (B) Tracking of PKH26 (red) labeled pMSCs and RPCs in the cryosections of the retina at 4 and 8 weeks. Scale bars represent 100 μm. (Magnification: ×20). (C) Graphical representation comparing the average thickness of the retina. Symbols, #, & and $ indicate significant difference at p ≤ 0.01 between all experimental conditions: rd12, rd12+pMSCs and rd12+RPCs, respectively

## Data Availability

The raw and processed sequencing data generated in this study have been deposited in the NCBI GenBank database, accession code PRJNA769116. All data needed to evaluate the conclusions in the paper are present in the paper and/or the Additional files 1, 2, 3.

## Abbreviations

**RDD**: Retinal degenerative diseases
**RP**: Retinitis pigmentosa
**MSCs**: Mesenchymal stem cells
**RPCs**: Retinal progenitor cells
**p**: Primitive
**AMD**: Age-related macular degeneration
**RPE**: Retinal pigment epithelium
**ESCs**: Embryonic stem cells
**iPSCs**: Induced pluripotent stem cell
**RCS**: Royal College of Surgeons
**BM**: Bone marrow
**h**: Human
**ERG**: Electroretinogram
**UC**: Umbilical cord
**WJ**: Wharton’s jelly
**RGC**: Retinal ganglion cells
**ONL**: Outer nuclear layer
**EGF**: Epidermal growth factor
**IACUC**: Institutional animal care and use committee
**IBC**: Institutional biosafety committee
**cpd**: Cycles per degree
**H&E**: Hematoxylin and eosin
**SEM**: Standard error of the mean
**DEGs**: Differentially expressed genes
**FDR**: False discovery rate
**GO**: Gene ontology
**INL**: Inner nuclear layer
**DIC**: Differential interface contrast
**HNA**: Human nuclear antigen

## References

[1] Hao X, Cheng J, Zhang Z (2018). Polymorphisms in PEDF linked with the susceptibility to age-related macular degeneration: a case-control study. Medicine (Baltimore)..

[2] O'Neal TB, Luther EE. Retinitis Pigmentosa. StatPearls. Treasure Island (FL): StatPearls Publishing Copyright © 2020, StatPearls Publishing LLC.; 2020.

[3] Surendran H, Rathod RJ, Pal R. Generation of Transplantable Retinal Pigmented Epithelial (RPE) Cells for Treatment of Age-Related Macular Degeneration (AMD). Methods in molecular biology (Clifton, NJ). 2018. 10.1007/7651_2018_140 29896658

[4] Hamel C (2006). Retinitis pigmentosa. Orphanet J Rare Dis..

[5] Marlhens F, Griffoin JM, Bareil C, Arnaud B, Claustres M, Hamel CP (1998). Autosomal recessive retinal dystrophy associated with two novel mutations in the RPE65 gene. Eur J Hum Genet.

[6] Prem Senthil M, Khadka J, Pesudovs K (2017). Seeing through their eyes: lived experiences of people with retinitis pigmentosa. Eye.

[7] Pang JJ, Chang B, Hawes NL, Hurd RE, Davisson MT, Li J (2005). Retinal degeneration 12 (rd12): a new, spontaneously arising mouse model for human Leber congenital amaurosis (LCA). Mol Vis.

[8] Cai X, Conley SM, Naash MI (2009). RPE65: role in the visual cycle, human retinal disease, and gene therapy. Ophthalmic Genet.

[9] Chaudhry GR, Fecek C, Lai MM, Wu W-C, Chang M, Vasquez A (2009). Fate of embryonic stem cell derivatives implanted into the vitreous of a slow retinal degenerative mouse model. Stem Cells Dev.

[10] Sun J, Mandai M, Kamao H, Hashiguchi T, Shikamura M, Kawamata S (2015). Protective effects of human iPS-derived retinal pigmented epithelial cells in comparison with human mesenchymal stromal cells and human neural stem cells on the degenerating retina in rd1 mice. Stem Cells.

[11] Zou T, Gao L, Zeng Y, Li Q, Li Y, Chen S (2019). Organoid-derived C-Kit(+)/SSEA4(-) human retinal progenitor cells promote a protective retinal microenvironment during transplantation in rodents. Nat Commun.

[12] Li Y, Tsai Y-T, Hsu C-W, Erol D, Yang J, Wu W-H (2012). Long-term safety and efficacy of human-induced pluripotent stem cell (iPS) grafts in a preclinical model of retinitis pigmentosa. Mol Med.

[13] Inoue Y, Iriyama A, Ueno S, Takahashi H, Kondo M, Tamaki Y (2007). Subretinal transplantation of bone marrow mesenchymal stem cells delays retinal degeneration in the RCS rat model of retinal degeneration. Exp Eye Res.

[14] Arnhold S, Absenger Y, Klein H, Addicks K, Schraermeyer U (2007). Transplantation of bone marrow-derived mesenchymal stem cells rescue photoreceptor cells in the dystrophic retina of the rhodopsin knockout mouse. Graefe's Arch Clin Exp Ophthalmol.

[15] Tzameret A, Sher I, Belkin M, Treves AJ, Meir A, Nagler A (2014). Transplantation of human bone marrow mesenchymal stem cells as a thin subretinal layer ameliorates retinal degeneration in a rat model of retinal dystrophy. Exp Eye Res.

[16] Wang S, Lu B, Girman S, Duan J, McFarland T, Zhang QS (2010). Non-invasive stem cell therapy in a rat model for retinal degeneration and vascular pathology. PLoS ONE..

[17] Qu L, Gao L, Xu H, Duan P, Zeng Y, Liu Y (2017). Combined transplantation of human mesenchymal stem cells and human retinal progenitor cells into the subretinal space of RCS rats. Sci Rep.

[18] Beeravolu N, McKee C, Alamri A, Mikhael S, Brown C, Perez-Cruet M, et al. Isolation and characterization of mesenchymal stromal cells from human umbilical cord and fetal placenta. J Vis Exp. 2017(122). 10.3791/55224 PMC5564456 28447991

[19] Mattiucci D, Maurizi G, Leoni P, Poloni A (2018). Aging- and senescence-associated changes of mesenchymal stromal cells in myelodysplastic syndromes. Cell Transplant.

[20] Harvey AR, Hu Y, Leaver SG, Mellough CB, Park K, Verhaagen J (2006). Gene therapy and transplantation in CNS repair: the visual system. Prog Retin Eye Res.

[21] Millán-Rivero JE, Nadal-Nicolás FM, García-Bernal D, Sobrado-Calvo P, Blanquer M, Moraleda JM (2018). Human Wharton's jelly mesenchymal stem cells protect axotomized rat retinal ganglion cells via secretion of anti-inflammatory and neurotrophic factors. Sci Rep.

[22] Leow SN, Luu CD, Hairul Nizam MH, Mok PL, Ruhaslizan R, Wong HS (2015). Safety and efficacy of human Wharton's jelly-derived mesenchymal stem cells therapy for retinal degeneration. PLoS ONE.

[23] Koh AE-H, Alsaeedi HA, Rashid MBA, Lam C, Harun MHN, Ng MH (2021). Transplanted erythropoietin-expressing mesenchymal stem cells promote pro-survival gene expression and protect photoreceptors from sodium iodate-induced cytotoxicity in a retinal degeneration model. Front Cell Dev Biol.

[24] Mohamed EM, Abdelrahman SA, Hussein S, Shalaby SM, Mosaad H, Awad AM (2017). Effect of human umbilical cord blood mesenchymal stem cells administered by intravenous or intravitreal routes on cryo-induced retinal injury. IUBMB Life.

[25] Zwart I, Hill AJ, Al-Allaf F, Shah M, Girdlestone J, Sanusi AB (2009). Umbilical cord blood mesenchymal stromal cells are neuroprotective and promote regeneration in a rat optic tract model. Exp Neurol.

[26] Labrador-Velandia S, Alonso-Alonso ML, Alvarez-Sanchez S, Gonzalez-Zamora J, Carretero-Barrio I, Pastor JC (2016). Mesenchymal stem cell therapy in retinal and optic nerve diseases: an update of clinical trials. World J Stem Cells.

[27] Tuekprakhon A, Sangkitporn S, Trinavarat A, Pawestri AR, Vamvanij V, Ruangchainikom M (2021). Intravitreal autologous mesenchymal stem cell transplantation: a non-randomized phase I clinical trial in patients with retinitis pigmentosa. Stem Cell Res Ther.

[28] Satarian L, Nourinia R, Safi S, Kanavi MR, Jarughi N, Daftarian N (2017). Intravitreal injection of bone marrow mesenchymal stem cells in patients with advanced retinitis pigmentosa; a safety study. J Ophthalmic Vis Res.

[29] Oner A, Gonen ZB, Sinim N, Cetin M, Ozkul Y (2016). Subretinal adipose tissue-derived mesenchymal stem cell implantation in advanced stage retinitis pigmentosa: a phase I clinical safety study. Stem Cell Res Ther.

[30] Weiss JN, Benes SC, Levy S (2016). Stem Cell Ophthalmology Treatment Study (SCOTS): improvement in serpiginous choroidopathy following autologous bone marrow derived stem cell treatment. Neural Regen Res.

[31] Weiss JN, Levy S, Benes SC (2016). Stem Cell Ophthalmology Treatment Study (SCOTS): bone marrow-derived stem cells in the treatment of Leber's hereditary optic neuropathy. Neural Regen Res.

[32] Weiss JN, Levy S, Benes SC (2017). Stem Cell Ophthalmology Treatment Study: bone marrow derived stem cells in the treatment of non-arteritic ischemic optic neuropathy (NAION). Stem cell Investig.

[33] Liu Y, Chen SJ, Li SY, Qu LH, Meng XH, Wang Y (2017). Long-term safety of human retinal progenitor cell transplantation in retinitis pigmentosa patients. Stem Cell Res Ther.

[34] Zhao T, Liang Q, Meng X, Duan P, Wang F, Li S (2020). Intravenous infusion of umbilical cord mesenchymal stem cells maintains and partially improves visual function in patients with advanced retinitis pigmentosa. Stem Cells Dev.

[35] Brown C, McKee C, Bakshi S, Walker K, Hakman E, Halassy S (2019). Mesenchymal stem cells: cell therapy and regeneration potential. J Tissue Eng Regen Med.

[36] Beeravolu N, Khan I, McKee C, Dinda S, Thibodeau B, Wilson G (2016). Isolation and comparative analysis of potential stem/progenitor cells from different regions of human umbilical cord. Stem Cell Res.

[37] Ye J, Coulouris G, Zaretskaya I, Cutcutache I, Rozen S, Madden TL (2012). Primer-BLAST: A tool to design target-specific primers for polymerase chain reaction. BMC Bioinform.

[38] McKee C, Brown C, Bakshi S, Walker K, Govind CK, Chaudhry GR (2021). Transcriptomic analysis of Naïve human embryonic stem cells cultured in three-dimensional PEG scaffolds. Biomolecules.

[39] Blankenberg D, Hillman-Jackson J (2014). Analysis of next-generation sequencing data using Galaxy. Methods Mol Biol.

[40] Mi H, Muruganujan A, Casagrande JT, Thomas PD (2013). Large-scale gene function analysis with the PANTHER classification system. Nat Protoc.

[41] Xie Z, Bailey A, Kuleshov MV, Clarke DJB, Evangelista JE, Jenkins SL (2021). Gene set knowledge discovery with enrichr. Curr Protoc.

[42] Molday LL, Cheng CL, Molday RS (2019). Cell-specific markers for the identification of retinal cells and subcellular organelles by immunofluorescence microscopy. Methods Mol Biol.

[43] Nicoletti A, Wong DJ, Kawase K, Gibson LH, Yang-Feng TL, Richards JE (1995). Molecular characterization of the human gene encoding an abundant 61 kDa protein specific to the retinal pigment epithelium. Hum Mol Genet.

[44] Lalitha S, Basu B, Surya S, Meera V, Riya PA, Parvathy S (2020). Pax6 modulates intra-retinal axon guidance and fasciculation of retinal ganglion cells during retinogenesis. Sci Rep.

[45] Gao HM, Hong JS (2008). Why neurodegenerative diseases are progressive: uncontrolled inflammation drives disease progression. Trends Immunol.

[46] Baranov PY, Melo GB, Young MJ (2012). Characterization of human retinal progenitor cells. Invest Ophthalmol Vis Sci..

[47] Schmitt S, Aftab U, Jiang C, Redenti S, Klassen H, Miljan E (2009). Molecular characterization of human retinal progenitor cells. Invest Ophthalmol Vis Sci.

[48] Wang J, Yang J, Gu P, Klassen H (2010). Effects of glial cell line-derived neurotrophic factor on cultured murine retinal progenitor cells. Mol Vis.

[49] Fu L, Hu Y, Song M, Liu Z, Zhang W, Yu F-X (2019). Up-regulation of FOXD1 by YAP alleviates senescence and osteoarthritis. PLoS Biol..

[50] Diehn JJ, Diehn M, Marmor MF, Brown PO (2005). Differential gene expression in anatomical compartments of the human eye. Genome Biol..

[51] Gipson CD, Olive MF (2017). Structural and functional plasticity of dendritic spines—Root or result of behavior?. Genes Brain Behav.

[52] Zhang Y, Harrison JM, Nateras OS, Chalfin S, Duong TQ (2013). Decreased retinal-choroidal blood flow in retinitis pigmentosa as measured by MRI. Doc Ophthalmol Adv Ophthalmol.

[53] Wang N-K, Tosi J, Kasanuki JM, Chou CL, Kong J, Parmalee N (2010). Transplantation of reprogrammed embryonic stem cells improves visual function in a mouse model for retinitis pigmentosa. Transplantation.

[54] Becker S, Eastlake K, Jayaram H, Jones MF, Brown RA, McLellan GJ (2016). Allogeneic transplantation of Müller-derived retinal ganglion cells improves retinal function in a feline model of ganglion cell depletion. Stem Cells Transl Med.

[55] Hasegawa T, Ikeda HO, Nakano N, Muraoka Y, Tsuruyama T, Okamoto-Furuta K (2016). Changes in morphology and visual function over time in mouse models of retinal degeneration: an SD-OCT, histology, and electroretinography study. Jpn J Ophthalmol.

[56] Bakondi B, Girman S, Lu B, Wang S (2017). Multimodal delivery of isogenic mesenchymal stem cells yields synergistic protection from retinal degeneration and vision loss. Stem Cells Transl Med.

[57] Ren G, Su J, Zhang L, Zhao X, Ling W, L'Huillie A (2009). Species variation in the mechanisms of mesenchymal stem cell-mediated immunosuppression. Stem Cells.

[58] Li XX, Yuan XJ, Zhai Y, Yu S, Jia RX, Yang LP (2019). Treatment with stem cells from human exfoliated deciduous teeth and their derived conditioned medium improves retinal visual function and delays the degeneration of photoreceptors. Stem Cells Dev.

[59] Kim GH, Paik SS, Park YS, Kim HG, Kim IB (2019). Amelioration of mouse retinal degeneration after blue LED exposure by glycyrrhizic acid-mediated inhibition of inflammation. Front Cell Neurosci.

[60] Das G, Choi Y, Sicinski P, Levine EM (2009). Cyclin D1 fine-tunes the neurogenic output of embryonic retinal progenitor cells. Neural Dev.

[61] Toops KA, Hagemann TL, Messing A, Nickells RW (2012). The effect of glial fibrillary acidic protein expression on neurite outgrowth from retinal explants in a permissive environment. BMC Res Notes.

[62] Ortuno-Lizaran I, Peterson DA, Cuenca N (2018). Neurogenesis detection in the adult human retina. Acta Ophthalmol.

[63] Xia X, Ahmad I (2016). Unlocking the neurogenic potential of mammalian Müller Glia. Int J Stem Cells.

[64] da Silva-Junior AJ, Mesentier-Louro LA, Nascimento-Dos-Santos G, Teixeira-Pinheiro LC, Vasques JF, Chimeli-Ormonde L (2021). Human mesenchymal stem cell therapy promotes retinal ganglion cell survival and target reconnection after optic nerve crush in adult rats. Stem Cell Res Ther.

[65] Vitallé J, Terrén I, Orrantia A, Bilbao A, Gamboa PM, Borrego F (2020). The expression and function of CD300 molecules in the main players of allergic responses: mast cells, basophils and eosinophils. Int J Mol Sci.

[66] Miczán V, Kelemen K, Glavinics JR, László ZI, Barti B, Kenesei K (2021). NECAB1 and NECAB2 are prevalent calcium-binding proteins of CB1/CCK-positive GABAergic interneurons. Cereb Cortex.

[67] Paylakhi S, Labelle-Dumais C, Tolman NG, Sellarole MA, Seymens Y, Saunders J (2018). Müller glia-derived PRSS56 is required to sustain ocular axial growth and prevent refractive error. PLoS Genet..

[68] Baranzini SE, Srinivasan R, Khankhanian P, Okuda DT, Nelson SJ, Matthews PM (2010). Genetic variation influences glutamate concentrations in brains of patients with multiple sclerosis. Brain J Neurol.

[69] Özmert E, Arslan U (2020). Management of retinitis pigmentosa by Wharton's jelly-derived mesenchymal stem cells: prospective analysis of 1-year results. Stem Cell Res Ther.

[70] Vilela CAP, Messias A, Calado RT, Siqueira RC, Silva MJL, Covas DT (2021). Retinal function after intravitreal injection of autologous bone marrow-derived mesenchymal stromal cells in advanced glaucoma. Doc Ophthalmol Adv Ophthalmol.

