# Supplementary figures and images for: Human primitive mesenchymal stem cell-derived retinal progenitor cells improved neuroprotection, neurogenesis, and vision in rd12 mouse model of retinitis pigmentosa
Source: Stem Cell Res Ther. 2022 Apr 8;13:148. doi: 10.1186/s13287-022-02828-w (PMC8994263; doi:10.1186/s13287-022-02828-w)

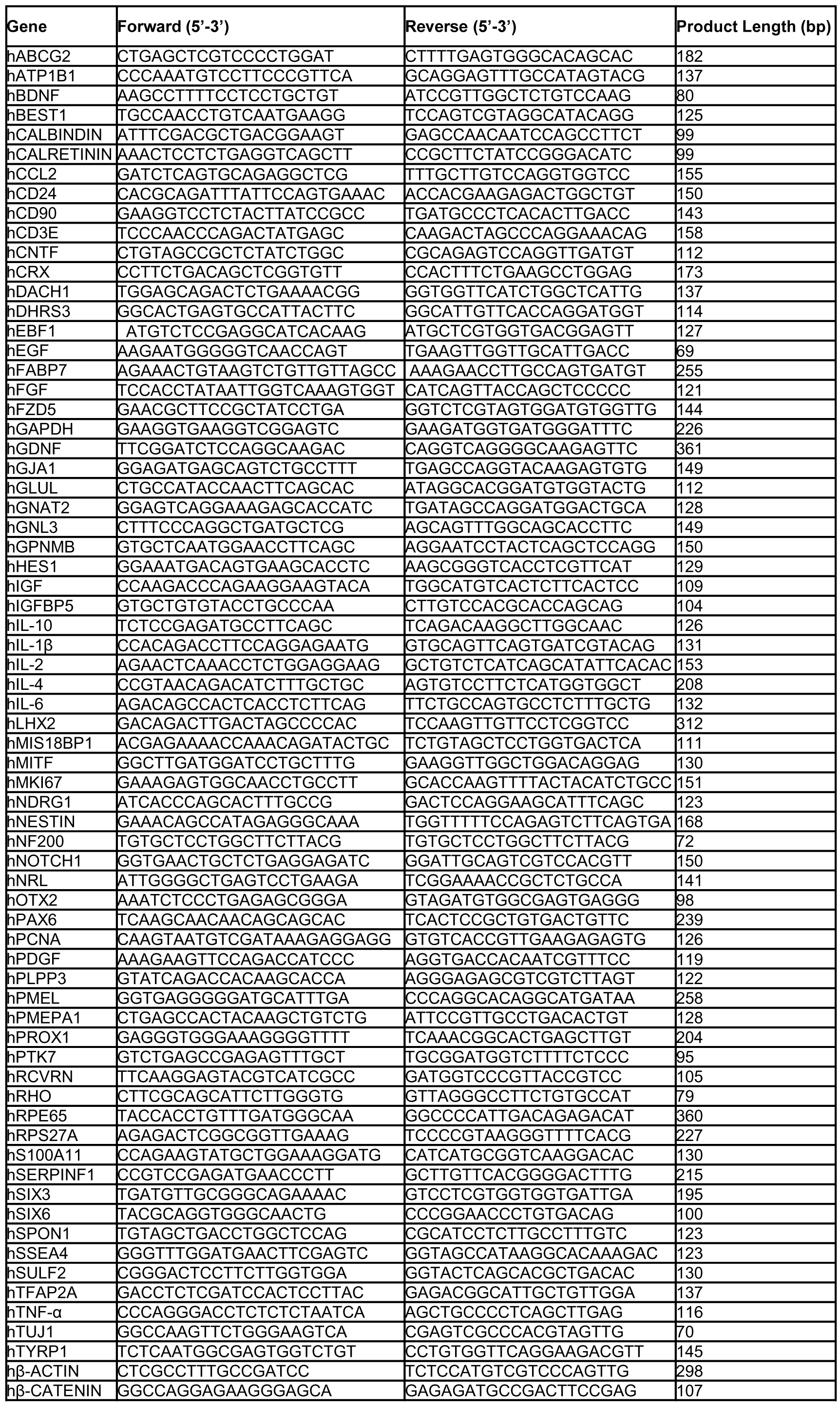

Supplement: Supplementary file 1 — Additional file 1. List of human primer sequences used in qRT-PCR [file 13287_2022_2828_MOESM1_ESM.tif]

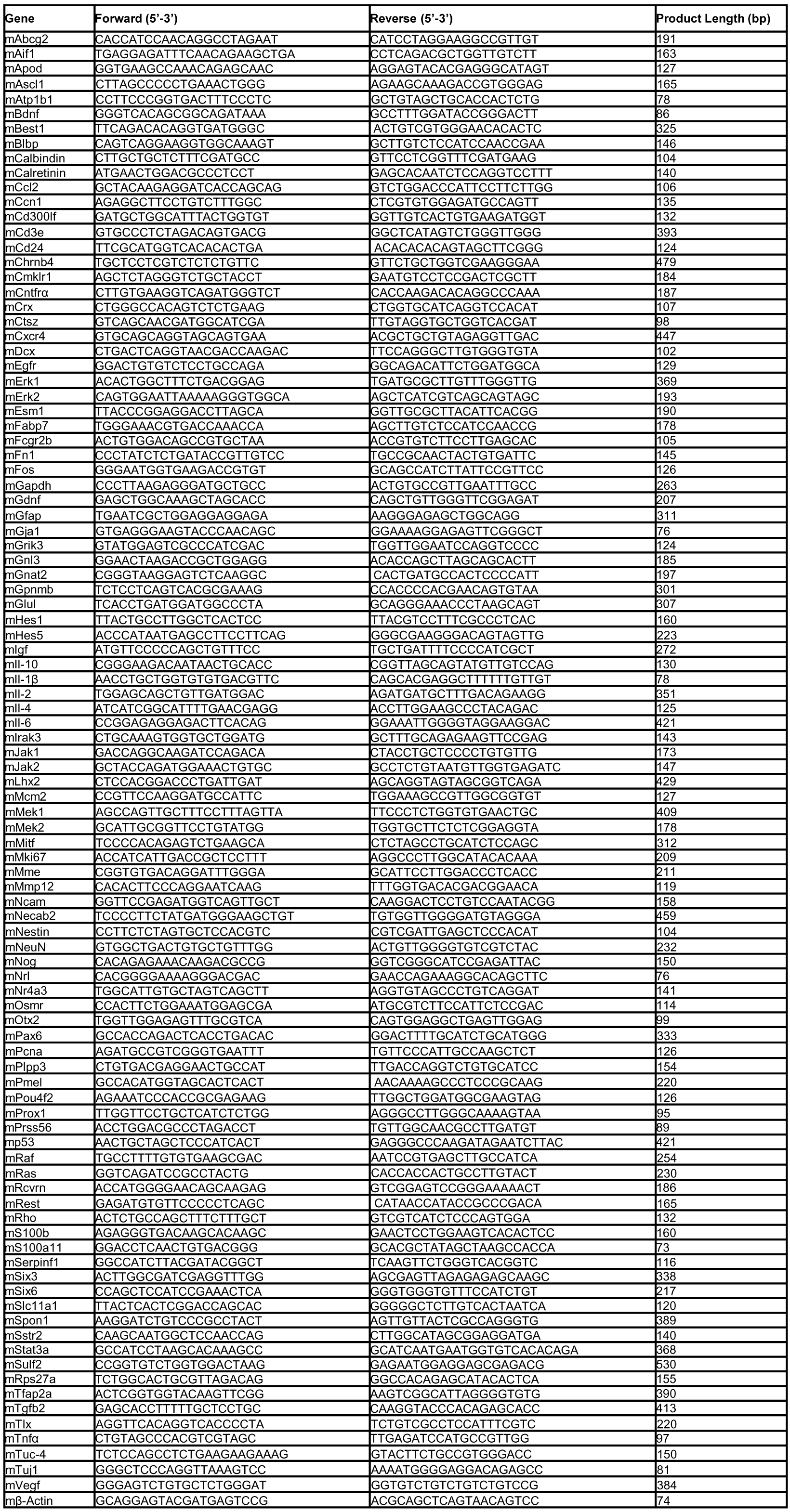

Supplement: Supplementary file 2 — Additional file 2. List of mouse primer sequences used in qRT-PCR [file 13287_2022_2828_MOESM2_ESM.tif]

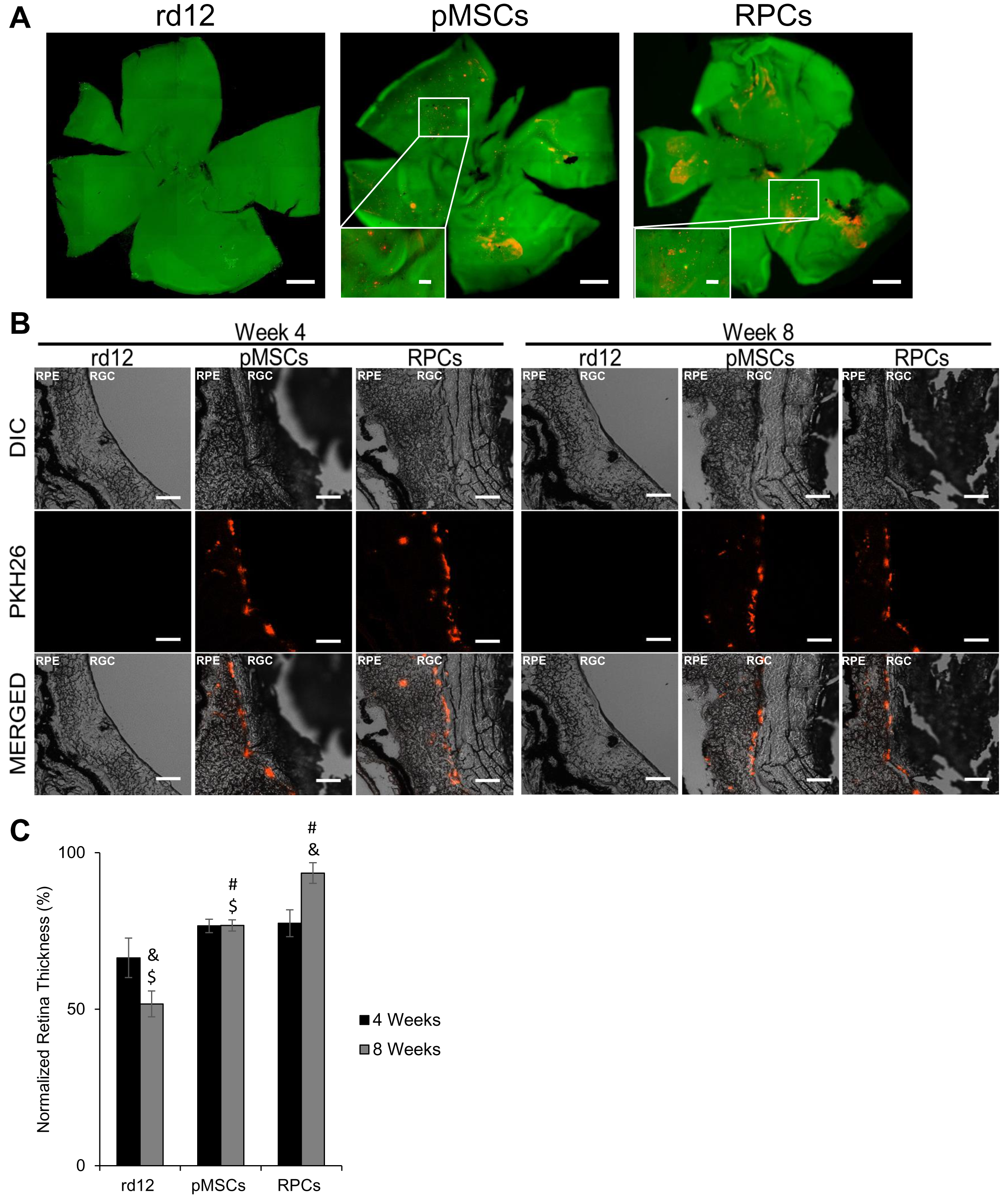

Supplement: Supplementary file 3 — Additional file 3. Tracking of cells transplanted into rd12 retina. (A) Whole-mount retina stained with RCVRN (green) 8 weeks after transplantation of PKH26-labeled (red) pMSCs and RPCs. 500 µm scale bar and 20 µm scale bars (inserts). (Magnification: ×5 and ×40, respectively). (B) Tracking of PKH26 (red) labeled pMSCs and RPCs in the cryosections of the retina at 4 and 8 weeks. Scale bars represent 100 μm. (Magnification: ×20). (C) Graphical representation comparing the average thickness of the retina. Symbols, #, & and $ indicate significant difference at p ≤ 0.01 between all experimental conditions: rd12, rd12+pMSCs and rd12+RPCs, respectively [file 13287_2022_2828_MOESM3_ESM.tif]
